# Vanishing Boycott Impetus: Why and How Consumer Participation in a Boycott Decreases Over Time

**DOI:** 10.1007/s10551-021-04997-9

**Published:** 2021-11-24

**Authors:** Wassili Lasarov, Stefan Hoffmann, Ulrich Orth

**Affiliations:** 1 Department of Marketing, Faculty of Business, Economics and Social Sciences, Christian-Albrechts-Universität zu Kiel, Westring 425, 24118 Kiel, Germany; 2 Institute of Agricultural Economics, Faculty of Agricultural and Nutritional Sciences, Christian-Albrechts-Universität zu Kiel, Wilhelm-Seelig-Platz 6/7, 24098 Kiel, Germany

**Keywords:** Boycott, Dynamics, Perceived egregiousness, Hot/cool cognition

## Abstract

Media reports that a company behaves in a socially nonresponsible manner frequently result in consumer participation in a boycott. As time goes by, however, the number of consumers participating in the boycott starts dwindling. Yet, little is known on why individual participation in a boycott declines and what type of consumer is more likely to stop boycotting earlier rather than later. Integrating research on drivers of individual boycott participation with multi-stage models and the hot/cool cognition system, suggests a “heat-up” phase in which boycott participation is fueled by expressive drivers, and a “cool-down” phase in which instrumental drivers become more influential. Using a diverse set of real contexts, four empirical studies provide evidence supporting a set of hypotheses on promotors and inhibitors of boycott participation over time. Study 1 provides initial evidence for the influence of expressive and instrumental drivers in a food services context. Extending the context to video streaming services, e-tailing, and peer-to-peer ridesharing, Study 2, Study 3, and Study 4 show that the reasons consumers stop/continue boycotting vary systematically across four distinct groups. Taken together, the findings help activists sustain boycott momentum and assist firms in dealing more effectively with boycotts.

**Supplementary Information:**

The online version contains supplementary material available at 10.1007/s10551-021-04997-9.

## Introduction

In 2013, a TV documentary on the substandard work conditions of employees who were subcontracted by a leading e-tailer evoked strong reactions with consumers in Germany, many of whom decided to boycott the company (Spiegel.de, 2013). After a while, however, public outrage and boycott participation waned (NTV.de, 2013). This anecdotal example ties in with econometric reports obtained at a macro level that boycotts lose participants and momentum over time (Chavis & Leslie, 2009). To date, however, researchers have not yet adopted an individual perspective on boycott participation to analyze promotors and inhibitors over time. Does boycott participation decline because consumer aggrevation fades, because consumers continue disapproving the transgression but revert to old habits for the sake of convenience, or because they loose faith in their boycott making a difference? Activists as well as managers need insights into these questions to respond more adequately.

Table 1 provides an overview of extant research that has examined drivers of boycott participation at the micro level. The table shows that previous research has almost exclusively employed cross-sectional studies (e.g., Klein et al., 2004; Sen et al., 2001). In addition, extant studies focused on consumers joining—instead of exiting—a boycott, hereby leaving a gap in knowledge about factors influencing a consumer's decision to sustain rather than stop boycotting. Furthermore, Table 1 illustrates that only a few studies suggest a temporal variation in and a possible revision of boycott decisions. For example, Chavis and Leslie (2009) showed boycotting to cease after an eight-month period, with sales returning to pre-boycott levels. However, the study adopted an aggregate perspective on boycotting and did not account for changes in individual behavior including possible drivers. Similarly, Ettenson and Klein (2005) reported two cross-sectional studies with data obtained from independent samples at two points in time. While they found an extension of boycotting beyond a one-year timeline, their study design did not permit drawing inferences regarding possible changes in individual boycotting behavior. Hoffmann (2011) gives additional insight on temporal effects. By grouping participants according to the dates they entered the boycott, he explored why consumers *join* boycotts at different stages. The study did not, however, extend to further changes in boycotting. In summary, previous research did not analyze temporal changes in boycott participation at the individual level after the decision to join had been made, nor did researchers examine the factors that impact changes. From a practical perspective, determining why consumers sustain or stop boycotting will help companies deal with boycotts more appropriately, and will aid activists in sustaining boycotts and keeping momentum.

**Table 1:**
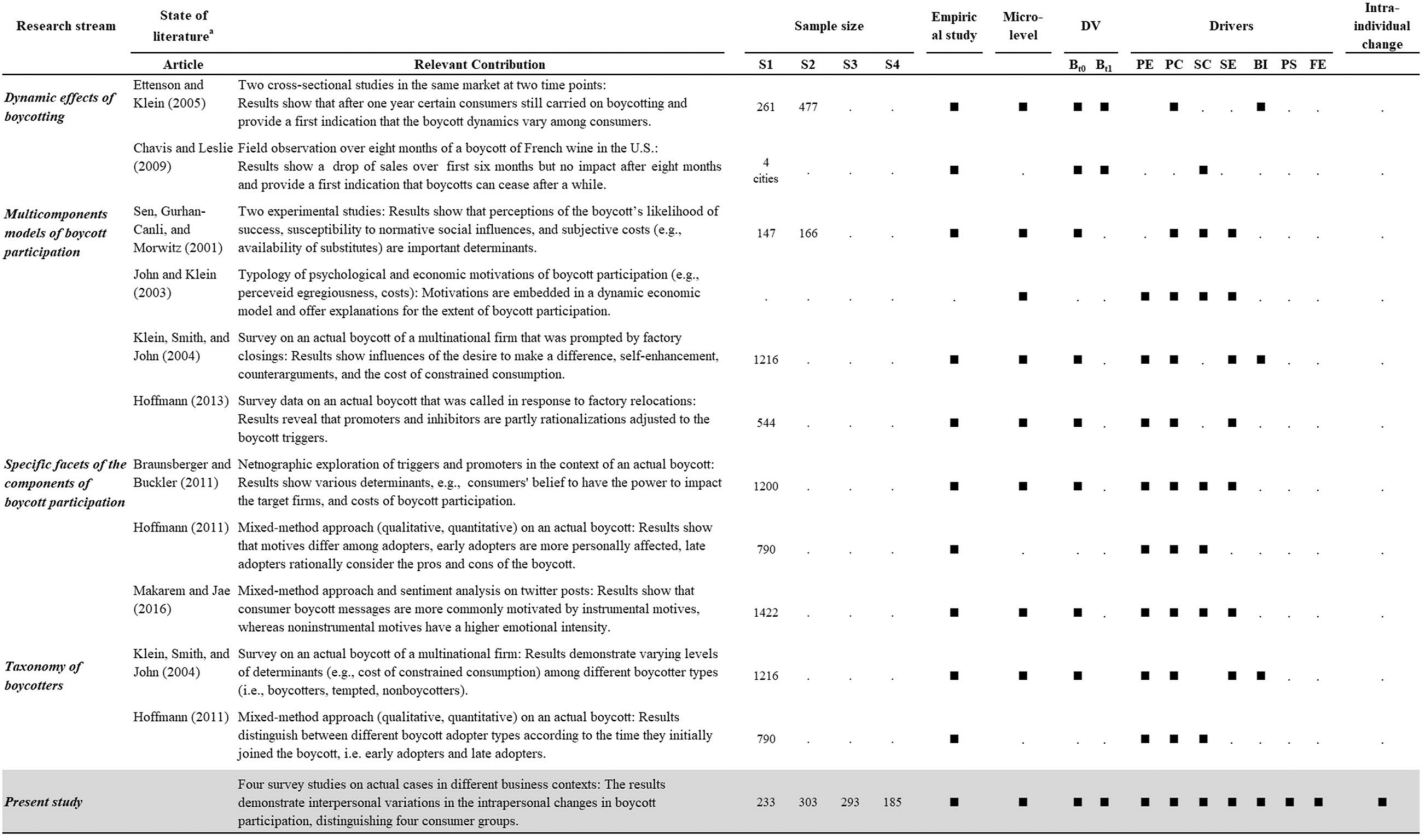
State of literature and contributions of this article

*PE *perceived egregiousness, *PC* perceived control, *SC* subjective costs, *SE* self-enhancement, *BI* brand image, *PS* perceived service quality, *FE* service of frontline employees, *DV* dependent variables, *B*_*t0*_ Boycott participation (*t*0), *B*_*t1*_ Boycott participation (*t*1)

^a^Only the papers that are most relevant to the current study are cited here. Only aspects related to the current study are documented

**Fig. 1:**
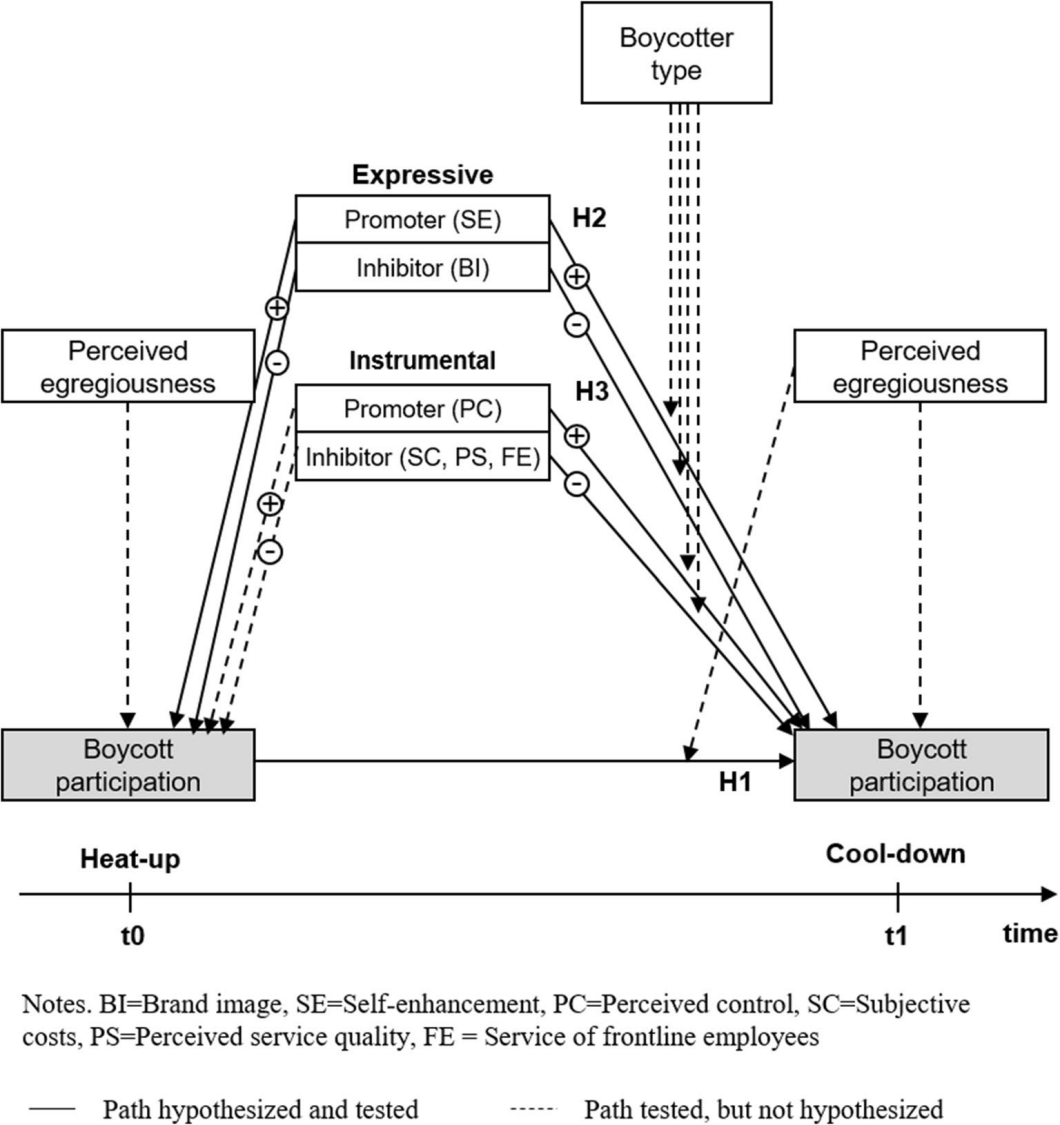
Conceptual model

Against this background, our study makes the following contributions to the literature (see Table 1). We extend boycott participation models (Hoffmann, 2011; Klein et al., 2004) to include a longer time period and to detail temporal changes in boycott participation at the individual consumer level. We label these changes "intrapersonal" to better communicate variations within an individual person across different points in time (Craik & Salthouse, 2008). Additionally integrated into the extension are consumer exits from the boycott, a perspective informed by research on the temporal effects of anger and revenge evoked by unethical behaviors (e.g., Ettenson & Klein, 2005; Klein et al., 1998; Lee et al., 2016; Sato et al., 2018). We extrapolate these findings to boycott contexts where the individual’s participation is driven by his or her perception of egregious conduct by the target firm (Klein et al., 2004). Although the perceived egregiousness contains both emotional and cognitive elements, social boycott calls often employ strongly emotional appeals, with moral condemnation of the target. Furthermore, by adopting Friedman’s (1999) distinction between expressive and instrumental boycotts we suggest a “heat-up” phase in which boycotters mainly make use of expressive drivers to join, and a “cool-down” phase in which additional instrumental drivers come into play, possibly causing a stop of boycotting. Finally, we identify distinct groups of consumers (boycotter types) who vary systematically in the reasons they continue and cease boycotting. Figure 1 illustrates the conceptual model underlying our research.

## Conceptual Background

### Boycott Participation: Definition and Extant Models

In his seminal article, Friedman (1985, p. 97) describes consumer boycotts as “… an attempt by one or more parties to achieve certain objectives by urging individual consumers to refrain from making selected purchases in the market place.” Activists have called consumer boycotts to achieve economic, social, ecological, ethical, ideological, or political objectives (Friedman, 1999; Sen et al., 2001) with regard to diverse issues including prices, human rights, working conditions, environmental protection, animal welfare, religion, or international politics (Yuksel et al., 2020). Boycotts can be direct or indirect (Friedman, 1999). In a direct boycott, participants avoid products and services of a target company whose policies they consider irresponsible. In an indirect boycott, participants avoid products of companies associated with a target, such as suppliers or firms located in a target country, to exert pressure on the target (Ettenson & Klein, 2005; Hoffmann et al., 2020).

In line with Friedman (1985), we view boycott participation as an individual consumer’s decision to respond to a collective call for a boycott by refraining from purchasing from a specific company or brand for the explicit purpose of achieving the boycott’s objectives. Importantly, this definition highlights that the participation supports a collective, group-driven action; it specifically excludes individualistic decisions to avoid brands (e.g., for reasons of personal health or identity). Further emphasizing group aspects, insights into consumer motivations of boycott participation (e.g., Klein et al., 2004; Sen et al., 2001) mainly utilize theories of social psychology and economics (e.g., theories of fairness and reciprocity, game theory, and social dilemma; Delacote, 2009; John & Klein, 2003). In this research stream, scholars have identified factors that drive consumers to join boycotts (e.g., self-enhancement), as well as factors that prevent them from boycotting (e.g., a lack of substitutes, inconvenience, skepticism about boycott efficacy; Klein et al., 2004; Sen et al., 2001).

Integrating and further detailing these factors, our research builds on and extends the model conceived by Klein et al. (2004) and refined by Hoffmann (2011). Klein et al.’s (2004) model views boycott participation as a deliberate act of abstinence. Initially, perceived egregiousness evokes arousal. Then, consumer boycott participation depends on anticipated rewards (such as self-enhancement) and the costs of abstaining from obtaining a preferred product. Hoffmann (2011) extended this model to include the “trigger/promoter/inhibitor” concept. We build on his conceptualization, because the trigger-promoter-inhibitor distinction is broader than the initial arousal-rewards-costs perspective, capturing a broader range of drivers of boycott participation. For example, while Klein et al.’s (2004) model includes only perceived egregiousness as a trigger of arousal, other studies show that a consumer’s proximity to the company’s wrongdoing can serve as an additional trigger (Hoffmann, 2011, 2013a). Furthermore, Klein et al.’s concept of benefits may be too narrow, as, for example, moral obligation can function as another promoter (Hoffmann et al., 2013b). Similarly, the original notion of costs may be too narrow, as other inhibitors, such as negative information about a competitor, have shown to be relevant (Yuksel & Mryteza, 2009). The trigger-promoter-inhibitor concept is therefore thought to be more flexible, accounting for additional and more divergent boycott participation motivations as identified in previous research.

### Triggers of Boycott Participation

According to Hoffmann (2011), the perception that a firm’s behavior is wrong triggers consumer behavioral response, because the perception negatively and harmfully affects workers, consumers, society at large, and other stakeholders. The extent to which the firm’s action is considered egregious depends on the individual. Accordingly, “perceived egregiousness” is the central trigger of boycott participation (Klein et al., 2004). Capturing the extent to which a person views an act (e.g., of a firm) as socially unacceptable, perceived egregiousness represents the level of a boycotter’s anger.

### Promoters of Boycott Participation

"Promoter" is an umbrella term used to capture factors that encourage boycott participation, specifically instrumental and moral factors (Hoffmann, 2011). Regarding instrumental factors, consumers are more likely to participate in a boycott when they expect their participation to increase the boycott's success (Sen et al., 2001), a type of boycott-related self-efficacy (Bandura, 2012). Regarding moral factors, consumers strive to enhance their self-esteem, and participating in a boycott—as a moral act—helps them do so (Klein et al., 2004). We therefore focus on perceived control and self-enhancement as important instrumental and moral promoters.

### Inhibitors of Boycott Participation

Inhibitors are factors that impede boycott participation. In line with previous studies (Hoffmann, 2011; Klein et al., 2004), we examine a variety of costs that occur when individuals boycott companies. First, withholding consumption is strongly associated with subjective costs, which, in turn, greatly depend on the availability of alternatives (Friedman, 1999; Sen et al., 2001). When consumers join a boycott, they may face costly challenges, such as gathering additional information about alternatives, abstaining from products they have preferred in the past, switching to more expensive alternatives, paying greater procurement costs, or even facing a complete lack of alternatives. While these subjective costs predominantly refer to increasing information costs, research costs, and financial costs involved in switching to other brands (or the lack of alternatives), there are other inhibitors that reflect other types of costs. A positive image can buffer against consumers’ boycott participation. Increased levels of trust decrease consumers’ willingness to participate in a boycott (Hoffmann & Müller, 2009), as they would have to build similar levels of trust with another brand. When consumers have long-standing positive associations with the company, they are therefore less likely to react negatively in times of crises, such as a transgression (Klein & Dawar, 2004). Consistent with this line of thought, a consumer’s overall satisfaction with the company and his or her positive experience from interactions with company employees might also increase switching costs and prevent him or her from boycotting.

### Developing a Model of Intrapersonal Variation in Boycott Participation

#### Intrapersonal Variation Moderated by Perceived Egregiousness

The previously discussed models of boycott participation have been limited to examining consumer motivations to boycott at one point in time. Suggesting that a temporal extension is needed, macro level studies indicate that boycotts gradually lose participants and momentum over time (Chavis & Leslie, 2009). Despite this overall decline in participation, consumers who were initially more determined to join the boycott (*t*0) may also be more likely to carry on boycotting during later stages (*t*1). Consistent with reports that initial egregiousness (*t*0) is a key driver of boycott participation in the initial phase (*t*0), possible changes in boycott participation should depend on perceived egregiousness (Klein et al., 2004). Although temporal aspects have not been analyzed just yet, perceived egregiousness should decrease when the transgression trigger becomes less salient. This thinking is in accordance with the agenda-setting theory (McCombs, 2013; McCombs et al., 1998), which posits that media reports exert a major influence on the proportion of emphasis placed on news. Consequently, a topic’s salience should depend greatly on media coverage, with public attention diminishing over time as awareness shifts to other topics (McCombs & Shaw, 1972). In a boycotting context, consumers’ negative emotions should cool down as media reports of the transgression cease and as levels of perceived egregiousness decline. Although the degree of perceived egregiousness at a later time (*t*1) may influence boycott participation at that point in time (*t*1), the decision should further depend on the consumer’s initial decision to (not) join the boycott (*t*0). By partially replicating studies on perceived egregiousness as a key driver of boycott participation (Klein et al., 2004) and by adding a dynamic perspective, we expect participation in a boycott at *t*1 to depend on the interplay between the consumer’s initial boycott participation (*t*0) and his or her current level of perceived egregiousness (*t*1).

##### H1

Perceived egregiousness will moderate the relationship between the initial boycott participation and participation at a later point in time. The higher the perceived egregiousness at *t*1 is, the stronger the influence of the initial boycott participation (*t*0) on later boycott participation (*t*1) is.

#### Distinguishing Between Instrumental and Expressive Drivers

At the macro level, boycotts can be categorized as instrumental or expressive (Friedman, 1999).^1^Instrumental boycotts aim at forcing the target to change its action. Expressive boycotts, in contrast, serve to vent the participants’ frustration and displeasure with the target’s actions. We extend this conceptualization from the macro to the individual level to distinguish between expressive and instrumental drivers of boycotting. At the level of individual boycott decisions, Friedman’s (1999) categorization is in accordance with Hoffmann’s (2011) distinction between moral and instrumental factors. We use the term "expressive" instead of “moral,” because it is the broader concept and includes moral factors.

We view promoters and inhibitors of boycott participation as instrumental when they relate to a consumer’s deliberate evaluation of whether or not the boycott will be successful and what sacrifices would have to be made. Perceived control is categorized an instrumental promoter, because consumers should be motivated more to join when they expect that their participation will increase the boycott’s chance to succeed (Hoffmann, 2011; Klein et al., 2004). Subjective costs, such as higher costs for substitutes of the boycotted product or service, are categorized an instrumental inhibitor.

In contrast, expressive influences are driven more by affect and emotion than by deliberation. For example, enhancing one’s self-view by supporting a boycott's good cause constitutes an expressive driver. Driven by the anticipated emotion to feel good, self-enhancement differs from the rational evaluation of whether or not the boycott will be successful. Similarly, a brand's positive image captures an emotional attachment to the brand that inhibits boycott participation (Hoffmann & Müller, 2009). Brand image therefore reflects the emotional element of switching costs. While contrasting cognitive (cool) against emotional (hot) processes helps better structuring the divergent temporal dynamics of boycott drivers, readers should be cautioned that the distinction is not a hard and clear-cut one: For example, subjective costs tend to be more cognitive but can include emotional aspects (e.g., consumers do not want to boycott a brand they are attached to), whereas brand image tends to be more emotional but can additionally include cognitive aspects (e.g., expectations regarding a particular product or service quality delivered by the brand).

#### The Role of Instrumental and Expressive Drivers at Different Points in Time

Consumer researchers commonly distinguish between "hot" and "cold" cognitions as influencers of behavioral response (e.g., Madrigal, 2008). Hot cognition is a less conscious, quick, and automatic decision process often operationalized as emotion, whereas cold cognition is regarded as a fact-based conscious process usually operationalized as cognition (Madrigal, 2008; Metcalfe & Mischel, 1999). Linking the hot/cool system with expressive and instrumental drivers of boycott participation, consumers respond to transgressions with negative emotions like anger and contempt (e.g., in sports contexts, Lee et al., 2016). In this context, cognition relates to people judging the responsibility of a transgressor based on object-relevant interpretations (Coombs & Holladay, 2002). Integrating both pathways, hot and cold cognitions conspire to influence peoples’ response to transgressions (e.g., Sato et al., 2018). Differences in the temporal dynamics of hot and cold cognition are further important to the present context (Metcalfe & Mischel, 1999). Specifically, research on moral decision making points at an “emotion-then-deliberation” sequence where moral decisions are the result of initial emotional response, which can later be overridden by deliberate judgment (Evans, 2008; Haidt, 2001). Lastly, a diminishing importance of hot versus cold drivers ties in with research on service failure recovery (e.g., Tsarenko & Tojib, 2011), brand transgressions, and product-harm crisis (e.g., Khamitov et al., 2020), indicating that—as time progresses—customers become less emotional in dealing with the incident, moving from spontaneous emotional responses to more in-depth assessments and evaluations.

Consistent with this line of thought, we expect that the roles played by expressive and instrumental drivers in a consumer's decision to participate in a boycott will vary between earlier and later stages of the boycott. Specifically, while the decision to join a boycott may initially be driven more by emotion (i.e., by expressive factors), instrumental factors should become more influential over time, thereby leading to cognitive dissonance in the evaluation of the boycott and ultimatively to changes in the participation (Hinojosa et al., 2017). This notion ties in with findings that the motives for joining or abstaining from a boycott can vary over time (Hoffmann, 2011): Early boycotters tend to decide impulsively and act spontaneously, whereas consumers who enter the boycott at a later stage are more likely to account for the costs of constrained consumption.

As a theoretical underpinning of the shifting role of different drivers over the course of time, we build on and adapt the multi-stage model of organic consumption (Mai et al., 2021). According to this model, consumers join organic consumption for ecological and social reasons—both representing expressive drivers. For maintaining organic consumption over time, however, expressive factors become less relevant or even exert negative influences, as they incur subjective costs without providing individual benefits. In contrast, self-related benefits constituting more instrumental drivers are key to sustaining organic consumption over a longer time span (Mai et al., 2021). Cognitive and instrumental drivers therefore become more relevant as time passes. While the multi-stage model of organic consumption has been initially conceived to explain organic consumption, transferring it to a boycotting (i.e., non-consumption) context suggests that expressive drivers should be particularly relevant at the onset of the boycott, while instrumental drivers should be more relevant to sustain or cease boycott participation.

#### Emotional Heating

Expressive drivers should be especially relevant at the onset of a boycott when the decision to participate is largely based on emotional and impulsive drivers, with less attention given to more instrumental aspects, such as the boycott’s anticipated impact. Prominent among the well-established expressive drivers are self-enhancement and brand image (see Table 1). Self-enhancement represents a process whereby individuals “strive systematically to promote the perception that others think well of them” (Swann et al., 1989, p. 782). Building on the literature about helping behavior, Klein et al. (2004) argued that participating in a boycott for moral reasons with the intention to help those who suffer from the offending company's behavior, can promote the perception of the boycotter in the eyes of others. Self-enhancement therefore refers to a person’s belief that boycotting is the morally right thing to do; it also captures the notion that supporting a just cause can lead consumers to feel better about themselves, reducing feelings of guilt (Braunsberger & Buckler, 2011; Klein et al., 2004). By participating in a boycott and by associating themselves with people (boycotters) who act for a just cause, consumers boost their self-esteem. This line of thinking is consistent with reports that self-enhancement encourages boycott participation (Braunsberger & Buckler, 2011; Hoffmann, 2011, 2013a; Klein et al., 2004) and drives expressive customer behavior. We thus view self-enhancement as an expressive promoter, which should exert a positive influence both at the onset, as well as during later stages of a boycott.

##### H2a

As an expressive promoter, self-enhancement will influence boycott participation positively (i) at the initial stage (*t*0) and also (ii) at later stages (*t*1).

Representing an expressive inhibitor (Hoffmann & Müller, 2009), brand image includes emotional aspects, which are attributable to marketing activities, context variables, and perceiver characteristics. Due to long-standing positive associations, customers who think positively about a brand or firm tend to react less negatively to product-harm crises (Klein & Dawar, 2004). Given the brand image's buffering capacity, this expressive driver should exert a negative influence during the emotional heat-up phase and should similarly be relevant at later stages.

##### H2b

As an expressive inhibitor, brand image will influence boycott participation negatively (i) at the initial stage (*t*0) and also (ii) at later stages (*t*1).

#### Cognitive Cooling

Construal level theory (Trope & Liberman, 2003) posits that the psychological distance between a referent and a person impacts processing. Psychological distance, including temporal distance, influences abstract versus concrete thinking in terms of high-level versus low-level construals (Trope & Liberman, 2003). Corresponding to greater distance, high-level construals are more abstract and generalized mental representations. Low-level construals, in contrast, correspond to greater proximity (lesser distance); they are more detailed and concrete mental representations (Nussbaum et al., 2003).

Among the drivers discussed in the literature (see Table 1 for an overview), perceived control can be considered an instrumental promoter, as consumers deliberate whether boycotting will effectively change the company’s behavior. As individuals gain more insights into the boycott’s consequences, they are more likely to assess their own role and impact. In contrast to the influence of expressive drivers, deliberate thinking, as well as the systematic processing of arguments for and against boycotting (which may change over time), should therefore shape the impact of instrumental drivers. Since cognitive processing and the search for relevant information will take more time than spontaneous affective responses, instrumental drivers should exert their positive influence on boycott partipation more at later stages than at the onset of the boycott.

##### H3a

As an instrumental promoter, perceived control will influence boycott participation positively at later stages (*t*1).

We expect several instrumental inhibitors to influence boycott participation. Their specific effect may depend on the context, and we will therefore later test our model in a diverse set of contexts. Generally, instrumental inhibitors include subjective costs, perceived service quality, and customer-friendly behavior of frontline employees.

The subjective costs of boycotting can be viewed as an instrumental factor, because consumers commonly account for the subjective burdens associated with boycotting (Hoffmann & Müller, 2009). While subjective costs may be underestimated at the start of a boycott, individuals may later come to realize that continuing the boycott will require substantial investments in time and money. According to construal level theory (Trope & Liberman, 2003), individuals tend to overcommit to future tasks and events. As time passes, they often realize that they simply cannot complete all the tasks they had initially planned (Kahneman & Tversky, 1979). We thus expect that individuals may initially (at a higher-level construal) commit to a boycott due to expressive drivers. However, as times passes and construing boycotting becomes more concrete (e.g., when boycotters detail actual subjective costs and consequences, that is, lower-level construal), individuals may reconsider their initial decision. For example, a consumer may come to realize that they can no longer abstain from buying due to a lack of substitutes. Therefore, and possibly contrasting earlier outcomes, boycott-related subjective costs should become more influential as time passes.

##### H3b

As an instrumental inhibitor, subjective costs will influence boycott participation negatively at later stages (*t*1).

Our research builds on well-established constructs (perceived egregiousness, brand image, self-enhancement, perceived control, perceived costs) that have been validated in previous studies of boycotting. However, because our focus is on examining boycott dynamics across a number of divergent business contexts, relevant characteristics of these contexts need to be accounted for. For example, certain inhibitors may be particularly relevant in service contexts, especially inhibitors related to service intangibility and provider attributes (Zeithaml et al., 2006). We thus examine two industry-specific inhibitors thought to come into play after boycott participation started and which may even be more important than subjective costs. First, perceived service quality and the consumers’ overall satisfaction may be important in contexts like video-streaming, ride-pooling, and fast food restaurants (but not in e-commerce). In contrast, frontline employees and their capacity to keep consumers from boycotting may be more important in the context of fast food restaurants but not in the others.

Research on consumer response to questionable actions of service providers shows that certain consumers exhibit behavioral loyalty due to a perceived lack of adequate alternatives (Dick & Basu, 1994; Kumar & Shah, 2004). Boycotting a company would necessitate switching to an alternative provider. In those cases, the original company's perceived service quality correlates with subjective switching costs: The higher the service quality perception is, the higher are the subjective switching costs. The costs involved in searching for a substitute provider and the perceived risk involved in switching to a new provider determine the strength of a consumer's bond with a firm (Monroe, 1990; Zeithaml, 1988). We therefore expect that the higher the service quality is, the lower the likelihood of a boycott is.

##### H3c

As an instrumental inhibitor, perceived service quality will have a negative effect on boycott participation at later stages (*t*1).

Individuals vary in the importance placed on interpersonal and other service quality aspects (Driver & Johnston, 2001). In contexts where customers interact directly with employees, the behavior of these frontline employees may possibly exert a substantial influence on a customer’s boycott participation. In many cases, frontline employees represent a key touch point between the company and its customers (Hartline et al., 2000). The frontline employees' pivotal role can attenuate the negative effect of a scandal (e.g., Löhndorf & Diamantopoulos, 2014), suggesting that companies should actively employ frontline employees as a remedy, especially when they interact frequently with customers (von Walter et al., 2016). Moreover, frontline employees have the capacity to selectively and persuasively convey information to customers that might help the company overcome scandals (e.g., Bettencourt & Brown, 2003). They may assist in explaining their company’s response and reinforce messages in accordance with official communication (e.g., Jordan-Meier, 2011), or they may deliver better than average service quality after an egregious act. Given that consumers who received remedial communication from frontline employees after an ethical transgression give lesser weight to the transgression (Jones et al., 2011), we expect the following –

##### H3d

As an instrumental inhibitor, the customer-friendly behavior of frontline employees will have a negative effect on boycott participation at later stages (*t*1).

We tested our hypotheses in four empirical studies, using real cases from a variety of industries and contexts. Table 2 gives an overview and illustrates how the studies build on and extend each other in terms of contexts, management perspectives, samples, time lags, measures, and drivers of boycott participation.

**Table 2:** Flow of studies

Study	Industry	Management perspective	Country	Sample size	Time lag	Boycott measures		Drivers
1	Fast food restaurants	Product management	U.S	233	3 weeks	M (O, I)	M (O, I)	PE, PC, SE^a^, BI, FE
2	Entertainment	Employee management	U.S	303	2 weeks	M (B, O, I)	M (B, O, I)	PE, PC, SE, BI, PS
3	E-commerce	Employee management	GER	293	5 months	R (B, O, I)	M (B, O, I)	PE, PC, SE, BI, SC
4	Ride-sharing	Public relations	U.S	220	12 months	R (B, O, I)	M (B, O, I)	PE, PC, SE, BI, PS

Sample country of origin: U.S. = United States, GER = Germany

Boycott measures: *B* behavior, moral *O* Obligation (feeling morally obliged to boycott), *I* intention, *M* measured, *R* retrospectively indicated

Drivers of boycott participation: *PE* perceived egregiousness, *PC* perceived control, *SC* subjective costs, *SE* self-enhancement, *BI* brand image, *PS* perceived service quality, *FE* service of frontline employees

^a^Word-of-mouth-specific self-enhancement

## Study 1

As an initial test to our hypotheses, Study 1 examines intrapersonal changes in boycotting as influenced by perceived egregiousness. Study 1 also assesses the effects of instrumental, as well as expressive, promoters and inhibitors. Furthermore, the study tests the role of service quality as a possible buffer against boycotting and probes the capacity of frontline employees to attenuate negative effects (von Walter et al., 2016).

### Design

Applying a within-subjects design in a fast food context, with measurements taken at two points in time three weeks apart, Study 1 examined consumers' response to an actual case of questionable employee management. A media reports-based vignette informed participants about a leading fast food chain's questionable business practices and methods. Instructions highlighted that the presented reports were real news taken from a number of web sites. The company was portrayed as the target of a social media campaign following a politician’s statement that the company’s CEO received a total salary of $21.8 million, whereas average workers were paid only $7.00 per hour. Another news story centered on a billboard put up on New York’s Times Square on New Year’s Eve, calling attention to the suffering of chickens on farms supplying the fast food restaurant. The third story portrayed the company's employees, encouraged by the #MeToo movement, staging a one-day strike at restaurants in ten major cities to push management to take stronger action against on-the-job sexual harassment. Tying the three cases together, study participants were informed that customers started boycotting the company, switching to other fast food providers. The vignettes are displayed in Appendix A3.

We collected data at two points in time, with a time lag of three weeks.^2^ Recruited through MTurk, 632 U.S. residents initially took part in an online survey (M_age_ = 36.87, SD_age_ = 12.04; 55% male); 233 of them returned for the second set of measurements. Data sets from an additional 31 participants were subsequently dropped due to failing an attention check. We randomly recruited participants without screening for prior purchase of the company’s products. A non-response analysis showed no systematic differences between participants who completed both questionnaires and those who dropped out after the first round.^3^

In the first round, we started the survey by presenting the vignette, followed by the request to list three more questionable actions attributed to the brand. Since most subjects repeated the content from the vignettes, we could verify that the participants had no doubts about the realism and credibility of the study. Furthermore, listing further immoral aspects helped us not only rule out individual differences among the participants in terms of the evaluation of the company's immoral behaviors but also reinforce the intended effect of the vignettes. In the second round, to avoid priming bias we merely stated that this study would be a follow-up to the one they had completed previously. In both rounds, we assessed boycott behavior, as well as inhibitors and promoters. We adapted measures of boycott participation developed by Nerb and Spada (2001), as well as Sen et al. (2001). A similar approach was used for assessing perceived egregiousness (Klein et al., 2004) at two points in time. Measures of expressive drivers (brand image and self-enhancement (exit)) and instrumental drivers (perceived control and subjective costs) were adopted from Klein et al. (2004). Additional measures assessed service quality (Parasuraman et al., 1985, 1988). Lastly, we developed a new scale to ascertain the participants' satisfaction with the frontline employees' service. Following Hirschmann (1970), we use “self-enhancement (voice)” for the word-of-mouth-specific self-enhancement and “self-enhancement (exit)” for the boycott-specific self-enhancement. In addition to the established items for assessing general self-enhancement (Klein et al., 2004), we therefore included items to assess self-enhancement (voice) (Alexandrov et al., 2013). The results of an exploratory factor analysis (principal component analysis, oblimin rotation) yielded two factors corresponding to two distinct self-enhancement constructs, thus suggesting discriminant validity. To decrease drop-out rates, short scales were given preference (see Table A2 in the Web Appendix). Note that full scales were employed in Study 3 and Study 4.

### Results

All indicators were mean centered before computing interaction terms (Aiken et al., 1991; Cohen et al., 2003). First, we ran ordinary least squares (OLS) regressions with boycott partipation (*t*0), perceived egregiousness (*t*1), and the interaction term of both variables as the independent variables and boycott participation as the dependent variable (Table 3: model 1, model 4). We then incrementally added the instrumental and expressive determinants as they had been established in previous studies (Table 3: model 2, model 5). Lastly, we included two determinants deemed to be particularly relevant in service contexts (Table 3: model 3, model 6). In line with H1, results show a significant boycott participation (*t*0) × perceived egregiousness (*t*1) interaction effect on boycott participation (*t*1) (*p* = 0.021, Table 3, model 4). When adding promoters and inhibitors (models 2 and 5), inhibitor effects were significant and negative, whereas promoter effects were significant and positive. Consistent with H2a, self-enhancement (exit), an expressive promoter, influenced boycott participation positively at both points in time: *t*0 (*p* = 0.002, Table 3, model 5) and *t*1 (*p* = 0.045, Table 3, model 5). Partially supporting H2b, brand image, the expressive inhibitor, had significant negative effects in *t*0 (*p* = 0.002, Table 3, model 2) but no effects in *t*1 (*p* = 0.973, Table 3, model 5). Supporting H3a, perceived control, the instrumental promoter, had no effect on boycott participation in *t*0 (*p* = 0.081, Table 3, model 2) but a positive significant influence in *t*1 (*p* = 0.054, Table 3, model 5). When adding the set of service-related determinants, frontline employee service had a negative influence on boycott participation in *t*1 (Table 3, model 6), supporting H3d.

**Table 3:** Study 1: determinants of Boycott participation at *t*0 and *t*1

	Boycott participation *t*0									Boycott participation *t*1								
	Model 1			Model 2			Model 3			Model 4			Model 5			Model 6		
	*β*	*t*	*p*	*β*	*t*	*p*	*β*	*t*	*p*	*β*	*t*	*p*	*β*	*t*	*p*	*β*	*t*	*p*
*Controls*																		
Age	− 0.11	− 1.39	0.168	− 0.02	− 0.34	0.733	− 0.02	− 0.37	0.713	− 0.07	− 0.95	0.343	− 0.02	− 0.33	0.739	− 0.04	− 0.57	0.567
Gender^1^	− 0.03	− 0.37	0.709	− 0.05	− 0.83	0.407	− 0.05	− 0.82	0.416	− 0.03	− 0.46	0.648	0.03	0.43	0.665	− 0.01	− 0.15	0.884
Education^2^	− 0.07	− 0.89	0.377	− 0.13	− 2.12	0.036	− 0.13	− 2.11	0.037	− 0.08	− 1.21	0.228	− 0.13	− 1.97	0.051	− 0.13	− 1.85	0.067
Income^3^	− 0.10	− 1.23	0.222	− 0.08	− 1.36	0.176	− 0.09	− 1.36	0.175	− 0.03	− 0.46	0.648	− 0.03	− 0.47	0.636	0.00	0.07	0.946
*Boyoctt and Egregiousness*																		
Perceived egregiousness (*t*0)	0.61	7.63	0.000	0.11	1.24	0.216	0.12	1.23	0.222									
Boycott (*t*0)										0.40	4.74	0.000	0.12	0.98	0.331	0.13	1.10	0.273
Perceived egregiousness (*t*1)										0.43	4.88	0.000	0.30	3.22	0.002	0.29	3.09	0.003
Boycott (*t*0) × perceived egregiousness (*t*1)										0.17	2.35	0.021	0.11	1.55	0.124	0.16	2.07	0.042
*Boycott drivers*																		
Self-enhancement (exit)				0.36	3.21	0.002	0.36	3.04	0.003				0.28	2.03	0.045	0.32	2.16	0.033
Self-enhancement (voice)				0.09	1.39	0.167	0.09	1.38	0.170				0.07	0.85	0.400	0.07	0.90	0.372
Brand image				− 0.26	− 3.09	0.002	− 0.27	− 2.77	0.006				0.00	0.03	0.973	0.00	0.02	0.981
Perceived control				0.18	1.76	0.081	0.18	1.75	0.083				0.19	1.95	0.054	0.13	1.35	0.179
Subjective costs				0.15	1.90	0.060	0.15	1.88	0.062				0.07	0.90	0.372	− 0.04	− 0.40	0.688
Customer service							0.01	0.06	0.954							− 0.31	− ﻿1.71	0.090
Frontline employees							0.01	0.10	0.918							− 0.33	− 2.30	0.024
R^2^	0.39			0.57			0.57			0.53			0.60			0.62		
Adj. R^2^	0.36			0.52			0.52			0.50			0.55			0.56		

Ordinary least squares regression. ^1^dummy coded: 0 = female, 1 = male; ^2^dummy coded: 0 = €1500 or less; 1 = €1501 or more; ^3^dummy coded: 0 = high school diploma or lower; 1 = more than high school diploma

### Discussion

Our results show that the initial boycott participation (*t*0) interacts with perceived egregiousness at *t*1 to affect boycott participation at *t*1. Effects vary systematically for expressive drivers (perceived egregiousness and brand image) and also for instrumental drivers (subjective costs and perceived control); furthermore, effects vary between the heat-up and the cool-down phases. Study 1 also highlights the role of frontline employees in dealing with boycotts. Especially in business contexts where frontline employees contribute substantially to customers’ perception of the company, such as in fast food restaurants, perceived service quality overrides other influencers of boycott participation. This finding has strong practical implications, because increasing numbers of fast food chains implement self-order kiosks, thereby reducing direct contact between customers and employees. Our findings suggest that this approach can backfire in times of egregious acts because of a lack of opportunities for frontline employees to restore damaged customer relationships.

## Study 2

Study 2 builds on and extends Study 1 in three important respects. First, Study 2 partially replicates Study 1 and extends it to the context of video streaming to enhance validity. As a check to the model’s robustness we also employed a new measure for boycott participation. Second, the study aims to corroborate the crucial role of perceived service quality in a context characterized by lower-frequency customer-frontline employee contact (different from Study 1). Third, Study 2 further disentangles the roles played by boycott drivers with distinct consumer groups. We explore temporal changes in a within-subjects design at two points in time with a time lag of two weeks.

### Design

Study 2 examined how consumers respond to the alleged immoral behavior of a leading online movie streaming provider. Participants first read a vignette including news reports that the company offered morally questionable shows and movies. For example, a novel reality show format encouraged a group of actors to talk an unknowing participant into committing murder. In a second case, a woman blamed the firm for her daughter’s attempt to commit suicide. In a third case, a corporate comedian proudly announced that his show was the main catalyst for over 4,500 relationships being terminated, including many divorces. In light of these news, the vignette alleged that the company’s customers started to boycott the firm and to switch to other providers. Again, we explained to participants that the presented media excerpts presented real reports taken from actual news websites. Following the vignette, we again prompted participants to write down additional questionable behaviors of the firm. Again, subjects mostly repeated the content from the vignettes, indicating that they had no doubts about the realism and credibility of the study. The vignettes are displayed in Appendix A3.

We collected data at two points in time with a time lag of two weeks. Recruited from MTurk, 533 U.S. residents took part in an online survey (M_age_ = 34.78, SD_age_ = 10.31; 57% male), with 303 participants returning to continue at the second point in time. A non-response analysis indicated no significant differences between participants who completed the study at both times and those who dropped out after the first round.^4^

The overall design of the study was almost identical to that of Study 1, with all scales consisting of seven-point Likert-type ratings. Different from Study 1, we added a binary measure of boycott participation (“I will boycott company XY”; no = 0, yes = 1). To enhance the validity of the boycott scale, we included a behavioral measure by offering respondents an opportunity to participate in a lottery. Upon completing the survey, they could choose between four coupons, one of these valid with the video streaming company and three others valid with competitors. Choosing the coupon of the video streaming company was thought to indicate non-boycotting, while the other options were thought to indicate boycotting. Accordingly, the coupons provide a dichotomous index of boycotting vs. non-boycotting. The chi-square test with the dichotomous boycott measure and the dichotomous coupon choice indicated a significant relation between both variables (χ^2^(1) = 3.51, *p* = 0.043).

### Results

**Fig. 2:**
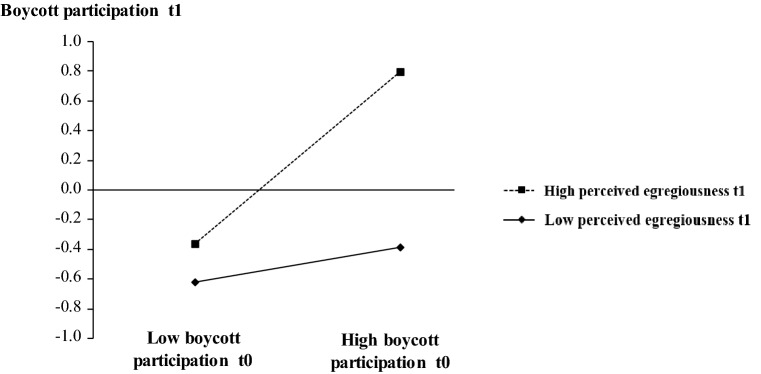
Interaction of boycott participation (*t*0) and perceived egregiousness (*t*1)

We ran binary logistic regressions with boycott participation (*t*0), perceived egregiousness (*t*1), and the interaction term of both variables as the independent variables, and a binary boycott participation measure as the dependent variable. All variables were mean centered (Aiken et al., 1991; Cohen et al., 2003). The variance inflation factor (VIF) indicates that multicollinearity does not bias our results (none of the VIFs reached a value above the threshold of 4; Hair et al., 2011). Results further indicate that—at *t*1—the interaction term between perceived egregiousness (*t*1) and boycott participation (*t*0) had a significant and positive effect (B = 0.41, Wald = 0.71, *p* ≤ 0.05) on boycotting (*t*1), thereby supporting H1. In other words, increasing levels of perceived egregiousness in *t*1 enhanced the initial boycott behavior's positive influence on boycott participation in *t*1. Figure 2 illustrates this finding.

Self-enhancement, the expressive promoter, influenced boycott participation significantly and positively in *t*0 (B = 0.27, Wald = 11.20, *p* ≤ 0.001), and had a marginally significant influence in *t*1 (B = 0.26, Wald = 2.86, *p* = 0.091). Similarly, brand image, the expressive inhibitor, had significant negative effects in both *t*0 (*B* = − 0.62, Wald = 4.20, *p* = 0.040) and *t*1 (*B* = 1.02, Wald = 4.28, *p* = 0.039). In contrast, perceived control, the instrumental promoter, had a marginally significant and positive effect on boycott participation only at *t*1 (*B* = 0.53, Wald = 2.93, *p* = 0.087). Among instrumental inhibitors, the effect of subjective costs on boycott participation was not significant at the two points in time, but perceived service quality had a significant negative influence in *t*1 only (*B* = − 0.82, Wald = 3.97, *p* = 0.046) as predicted in H3c.

Further extending Study 1, we gauged interpersonal differences in the temporal dynamics of boycott participation to enhance managerial implications. Specifically, we categorized respondents according to four distinct types: Respondents who exhibited an increasing boycott participation (*M*_*t*0_ = 2.67, *M*_*t*1_ = 3.93, *t* = 12.48, *p* ≤ 0.001) were labeled the *Deliberators* (Δboycott_*t*1_ − boycott_*t*0_ > 0). Respondents who exhibited constant levels (i.e., no significant difference between mean boycotting scores at the individual level) of boycott participation (M_*t*0,*t*1_ = 2.65) were labeled the *Apathetic*. To split the large group of remaining respondents exhibiting a decrease in boycott participation (Δboycott_*t*1_ − boycott_*t*0_ < 0), we additionally accounted for changes in the level of perceived egregiousness [(Δegregiousness < 0) or (Δegregiousness ≥ 0)]. Consumers who exhibited a decreasing boycott participation [*M*_*t*0_ = 4.66, *M*_*t*1_ = 3.00, *t* = 10.23, p ≤ 0.001) together with a decrease in perceived egregiousness (*M*_*t*0_ = 4.74, *M*_*t*1_ = 3.01, *t* = 11.39, *p* ≤ 0.001) were labeled the *Forgetters*. The last group, consisting of participants who exhibited a decrease in boycott participation (*M*_*t*0_ = 3.94, *M*_*t*1_ = 2.84, *t* = 10.40, *p* ≤ 0.001) combined with stable levels of egregiousness, was labeled the *Capitulated*. All respondents were assigned to one of the four groups. Next, we ran four separate regression models (one for each of the four groups), each with perceived egregiousness, promoters, and inhibitors as the independent variables, and boycott participation as the dependent variable (see Table 4, upper panel). The results indicate that self-enhancement has a strong effect on boycotting across types, making it little useful for explaining differences. In contrast, differences established for the remaining drivers fit our predictions. With the Deliberators, perceived control had a significant positive effect. Brand image had a strong negative effect with the Apathetic. Perceived egregiousness had a medium-sized effect with the Forgetters. For the Capitulated-type consumers, the instrumental inhibitor, subjective costs, was particularly relevant.

**Table 4:** Type-specific drivers of boycott participation in the cool-down phase (*t*1)

DV: boycott *t*1	The deliberators			The apathetic			The forgetters			The capitulated		
	*β*	*p*	*t*	*β*	*p*	*t*	*β*	*p*	*t*	*β*	*p*	*t*
*Study 2*												
Self-enhancement	0.09	0.134	1.54	0.10	0.020	2.47	0.08	0.011	2.05	0.10	0.043	2.63
Perceived control	0.36	0.048	2.06	0.39	0.006	2.95	0.33	0.464	3.09	0.09	0.003	0.74
Brand image	− 0.42	0.183	− 1.37	− 0.63	0.006	− 3.00	− 0.11	0.411	− 0.59	− 0.16	0.554	− 0.83
Perceived egregiousness (*t*1)	0.37	0.000	4.37	0.12	0.158	1.45	0.19	0.001	2.66	0.29	0.009	3.59
Subjective costs	0.02	0.656	0.45	0.09	0.049	2.05	0.05	0.069	1.51	0.07	0.133	1.65
Service quality	− 0.62	0.028	− 2.31	0.03	0.909	0.12	0.02	0.640	0.08	− 0.10	0.935	− 0.47
*R*^2^	0.72			0.81			0.72			0.50		
Adj. *R*^2^	0.66			0.77			0.69			0.45		
Share (in %)	15.4			15.0			27.7			41.9		
*Study 3*												
Self-enhancement	0.48	0.002	3.36	0.629	0.000	7.46	0.419	0.000	6.47	0.549	0.000	5.08
Perceived control	0.22	0.085	1.77	0.070	0.303	1.04	0.119	0.052	1.95	0.163	0.095	1.71
Brand image	− 0.11	0.300	− 1.05	− 0.337	0.000	− 4.97	− 0.229	0.000	− 3.82	− 0.157	0.097	− 1.70
Perceived egregiousness (*t*1)	0.19	0.187	1.34	0.064	0.363	0.91	0.284	0.000	4.87	0.291	0.011	2.69
Subjective costs	− 0.16	0.141	− 1.50	0.030	0.628	0.48	− 0.075	0.187	− 1.32	− 0.180	0.056	− 1.97
*R*^2^	0.60			0.80			0.56			0.70		
Adj. *R*^2^	0.54			0.78			0.54			0.65		
Share (in %)	13.3			19.8			51.5			15.4		
*Study 4*												
Self-enhancement	0.27	0.042	2.09	0.61	0.000	5.77	0.58	0.000	5.66	0.78	0.000	4.68
Perceived control	0.62	0.000	4.07	0.05	0.545	0.61	− 0.01	0.906	− 0.12	0.11	0.459	0.75
Brand image	− 0.20	0.083	− 1.77	− 0.05	0.418	− 0.82	− 0.15	0.121	− 1.57	0.06	0.527	0.64
Perceived egregiousness (*t*1)	0.05	0.605	0.52	0.36	0.001	3.48	0.31	0.003	3.09	0.02	0.876	0.16
Subjective costs	− 0.06	0.502	− 0.68	− 0.09	0.047	− 2.03	− 0.05	0.490	− 0.69	− 0.06	0.509	− 0.67
Service quality	− 0.07	0.042	− 0.73	0.10	0.179	1.36	− 0.02	0.857	− 0.18	− 0.27	0.021	− 2.44
R^2^	0.72			0.92			0.77			0.86		
Adj. R^2^	0.69			0.91			0.75			0.83		
Share (in %)	22.6			28.8			29.7			18.9		

Ordinary least squares regression

### Discussion

The findings of Study 2 corroborate the findings obtained in Study 1 in another business context, thus enhancing confidence in thegeneralizability of the findings. The findings of this study indicate that perceived egregiousness fades over time and that this effect is reflected in an overall decrease in boycott participation. Furthermore, this study shows that boycott participation varies over time between four distinct types of consumers due to the divergent influence of boycotting drivers.

## Study 3

Study 3 partially replicates previous studies and extends them to an e-tailing context. The study seeks to corroborate the inhibiting role of subjective costs that come into play when service-related factors (e.g., service quality, frontline employees) are muted due to the context. Different from the previous studies where promoters and inhibitors were measured with reduced scales (to lower drop-out rates due to respondent fatigue), Study 3 employs original (extended) scales of the constructs retrospectively assessed at a single point in time.

### Design

Study 3 focuses on an actual case in Germany where the misconduct of an e-retailer received extensive media coverage. In this case, German public television (TV) broadcast a documentary on the substandard work conditions of employees subcontracted by a leading online retailer. Watched by a large audience (2 million TV viewers plus another 2.4 million views online), this documentary evoked strong reactions with both the press (print and online) and society (especially in blogs and newspaper commentaries). Many consumers expressed their determination to boycott the company. In online postings, consumers stated they would feel ashamed to be seen purchasing products from the the e-tailer. Examining this case is especially appropriate, because, in boycotting, consumers make substantial sacrifices due to the e-tailer’s vast portfolio, its position as a market leader, and the overall convenience of buying online. As with previous studies, the stimuli highlighted that media reports were obtained from real online news.

Three hundred and five consumers were recruited using an online survey posted on various social networks. Ninety-nine point six percent of participants indicated to be familiar with the company, and 100% indicated being or having been customers. Twelve data sets were dropped due to incomplete information, leaving 293 respondents (*M*_age_ = 25.27, SD_age_ = 4.62, 63.5% female) for subsequent analyses.

First, we asked respondents to recall the documentary and aided their memory by reminding them of the key facts summarized in a short text. We specifically highlighted media reports that the company had lured more than 3,000 workers from abroad with offers of good pay and good working conditions, whereas actual salaries paid were much lower. Up to six workers had to share a small room and were under continuous video surveillance by a security firm suspected to have close ties with a German far-right party. Emphasizing these facts was intended to ascertain that consumers who already knew about the transgression were reminded of the relevant facts, whereas others who had not heard about it could develop a vivid and detailed image of the transgression (Spiegel.de 2013). The vignettes are displayed in Appendix A3.

Study participants next stated levels of perceived egregiousness and boycott participation for both the time of the survey (*t*1 = five months after the TV documentary) and, retrospectively, five months earlier (*t*0 = the time when the TV documentary was broadcast). Again, we adapted measures of boycott participation developed by Nerb and Spada (2001), as well as Sen et al. (2001). The items used to assess boycott participation at *t*0 were identical to the originals but phrased in the past tense (see Table A2 in the Web Appendix). We also captured facets of boycott participation, such as the perceived obligation to participate in a boycott.^5^ A similar approach was used for assessing perceived egregiousness (Klein et al., 2004) at two points in time. To increase validity, these questions appeared at the beginning of the questionnaire when the respondents were not yet aware of the study's possible objectives. Indicators of expressive drivers (brand image and self-enhancement) and instrumental drivers (perceived control and subjective costs) were adopted from Klein et al. (2004). All scales were of the seven-point Likert type.

Indicating sufficient reliability, Cronbach’s alpha for all multi-item scales exceeded the critical value of 0.70 (see Table A2 in the Web Appendix). Confirmatory factor analysis (maximum likelihood estimation, AMOS 24.0) of all multi-item drivers of boycott participation yielded an acceptable fit of the model (*χ*^2^/d.f. = 1.59, comparative fit index: CFI = 0.974, root mean square error of approximation: RMSEA = 0.045). Fornell and Larcker’s (1981) criterion provides evidence for the discriminant validity of these constructs; that is, the average variance extracted (AVE) for each construct is higher than the square of the correlation of this construct with any other construct (Table A1, Web Appendix). We ran the single-factor test of Harman (1967), which is the most commonly used post hoc approach to manage common method variance (CMV) (Fuller et al., 2016), to check whether CMV may bias the results.^6^ The first unrotated factor explains 27.5% of the indicators' shared variance, which is significantly less than the critical threshold of 50%. Taken together, the results suggest reliable and valid measures.

We also added a behavioral measure by offering respondents an opportunity to participate in a lottery. Upon completing the survey, they could choose between three coupons: a coupon valid with the boycotted firm, a second one valid with another online bookstore, and a third one valid with a local book store. The first option was thought to indicate non-boycotting, while—given the e-tailer's role as a market leader—the latter two options were considered to indicate boycotting. Accordingly, the coupons provide a dichotomous index of boycotting vs. non-boycotting. The correlation between the psychometric measure of boycott (*t*1) and the dichotomous variable of coupon choice was significant and negative (*r* = − 0.45, *p* ≤ 0.001).

### Results

As an initial test to our hypotheses, we examined changes in perceived egregiousness and boycott participation from *t*0 to *t*1. T-tests confirmed that both perceived egregiousness (*M*_*t*0_ = 5.90, *M*_*t*1_ = 5.14, *t* = 13.99, *p* = 0.007) and boycott participation (*M*_*t*0_ = 3.63, *M*_*t*1_ = 2.82, *t* = 12.58, *p* = 0.008) decreased significantly from the heat-up phase (*t*0) to the cool-down phase (*t*1).

We next ran OLS regressions to test hypotheses more directly (see Table 5), with all indicators mean centered before calculating interaction terms (Aiken et al., 1991; Cohen et al., 2003). The variance inflation factor indicates that multicollinearity does not bias our results.^7^ Again, the interaction term (see Table 5) between perceived egregiousness (*t*1) and boycott participation (*t*0) had a significant and positive effect on boycotting at *t*1.

**Table 5:** Study 3: determinants of Boycott participation at *t*0 and *t*1

	Boycott participation *t*0								Boycott participation *t*1							
	Model 1				Model 2				Model 3				Model 4			
	*β*	*p*	*t*	VIF	*β*	*p*	*t*	VIF	*β*	*p*	*t*	VIF	*β*	*p*	*t*	VIF
Controls																
Age	0.23	0.318	1.00	1.02	0.053	0.733	34	1.045	0.07	0.660	0.42	1.03	− 0.08	0.696	− 0.53	1.05
Gender^1^	0. -23	0.039	− 2.08	1.08	− 0.049	0.529	− 0.63	1.144	0.10	244	1.16	1.07	− 0.08	0.201	− 0.49	1.03
Income^2^	0.43	0.080	1.76	1.03	0.266	0.108	1.61	1.030	0.18	276	1.01	1.04	0.21	0.200	1.28	1.04
Education^3^	− 0.14.559	− 0.058	1.02	0.067	0.674	0.42	1.023	− 0.12	0.476	− 0.68	1.03	0.07	0.628	0.98	1.11	
Boycott and Egregiousness (*t*0)																
Perceived egregiousness(*t*0)	0.54	0.000	10.30	1.07	0.270	0.000	6.895	1.313								
Boycott (*t*1)									0.63	0.000	15.34	1.20	0.41	0.000	8.33	2.15
Perceived egregiouisness (*t*1)									0.34	0.000	8.44	1.13	0.27	0.000	7.39	1.19
Boycott (*t*0) × perceived egregiousness (tl)									0.10	0.006	2.81	1.02	0.08	0.010	2.47	1.03
Boycott driven Self-enhancement					0.541	0.000	1.23	1.707					0.23	0.002	5.14	1.87
Brand image					− 0.154	0.000	− 4.08	1.173					− 0.16	0.000	− 4.14	1.25
Perceived control					0.129	0.001	3.27	1.310					0.08	0.033	1.94	1.37
Subjective costs					− 0.036	0.301	4.04	1.068					− 0.12	0.001	− 3.42	1.07
*R*^2^	0.35				0.64				0.65				0.73			
Adj. *R*^2^	0.33				0.63				0.64				0.71			

Notes: Ordinary least squares regression. ^1^dummy-coded: 0 = female; 1 = male; ^2^dummy-coded: 0 = €1.500 or less; 1 = €1.501 or more; ^3^dummy-coded: 0 = high school diploma or loner; 1 = more than high school diploma. V1F = variance inflation factor

In line with expectations, inhibitors had negative and promoters had positive effects on boycott participation. Supporting H2a, the expressive promoter, self-enhancement, influenced boycott participation positively at the initial stage (see Table 5, Model 2) and at *t*1 (see Table 5, Model 4). Similarly, the expressive inhibitor brand image had significant negative effects at both points in time. The instrumental promoter, perceived control, had a positive effect on boycott participation at *t*1, supporting H3a. In support of H3b, the effect of the instrumental inhibitor, subjective costs, on boycott participation at *t*1 was significant and negative. Unexpectedly, perceived control had a significant and positive effect on boycott participation at *t*0.

As with Study 2, we categorized respondents according to the four boycotter types. *T*-tests confirm that the mean boycott participation for the *Deliberators* was higher in *t*1 than in *t*0 (*M*_*t*0_ = 2.85, *M*_*t*1_ = 3.44, *t* = 9.36, *p* ≤ 0.001). Respondents with a constant level of boycott participation over time (*M*_*t*0,*t*1_ = 2.48) were categorized as the *Apathetic*. Consumers who exhibited a decrease in boycott participation (*M*_*t*0_ = 4.16, *M*_*t*1_ = 2.69, *t* = 17.70, *p* ≤ 0.001) in combination with a decrease in perceived egregiousness (*M*_*t*0_ = 6.19, *M*_*t*1_ = 4.93, *t* = 16.78, *p* ≤ 0.001) were categorized the *Forgetters*. Consumers who exhibited a decrease in boycott participation (*M*_*t*0_ = 4.01, *M*_*t*1_ = 3.14, *t* = 9.39, *p* ≤ 0.001) combined with constant levels of perceived egregiousness (*M*_*t*0_ = 5.62, *M*_*t*1_ = 5.64, n.s.) were categorized as the *Capitulated*. An analysis (Chi-square test) of coupons chosen by the four consumer types indicated differences between the Forgetters (49% of which chose the e-tailer's coupon), the Capitulated (47%), and the Deliberators (51%), on the one hand, and the Apathetic (71%), on the other hand [*χ*^2^(3) = 9.02, *p* = 0.038]. As a check to the robustness of the results obtained with the psychometric measure, results with the behavioral measure corroborated that Apathetic consumers stay with the boycotted e-tailer more than do the other types.

To examine drivers of boycotting among the four types, we again ran four regression models (one for each type), with perceived egregiousness, promoters, and inhibitors as the independent variables, and boycott participation as the dependent variable (see Table 4, middle panel). Yet again, the results indicate a unique set of drivers for each of the four groups. With the Deliberators, only perceived control had a significant positive effect. Brand image had only a significant effect with the Apathetic consumers. Perceived egregiousness had a medium-sized effect with the Forgetters.

### Discussion

The findings of Study 3 corroborate results obtained with previous studies in a different context. Again, the findings indicate that perceived egregiousness fades over time and that this effect is reflected in an overall decrease in boycott participation. More specifically, initial boycott participation (*t*0) interacts with perceived egregiousness at *t*1 to affect boycott participation at *t*1. Furthermore, boycott participation over time varies between four types of consumers due to the divergent influence of boycotting drivers. Specifically, effects vary systematically for expressive drivers (perceived egregiousness and brand image), and also for instrumental drivers (subjective costs and perceived control). In addition, they vary between the heat-up and the cool-down phases. Furthermore, the results highlight the importance of socially responsible human resource management for employee work behaviors (Shen & Benson, 2016). Besides the direct negative influence on employee behaviors, public opinion could even be more damaging to the company if it violates certain standards of the working conditions. Despite the valuable insights provided, Study 3 had at least one limitation that motivated the final study: At the onset of the study, respondents were informed of key facts, irrespective of whether or not they actually recalled the original egregious act. The results could therefore have been biased, as certain respondents learned about the event for the first time.

## Study 4

Study 4 aims to address Study 3’s limitation and replicate the main findings of previous studies in yet another context. The study focuses on an actual boycott of a peer-to-peer ridesharing and transportation network firm, which became public knowledge 12 months prior to our study. As a methodological contribution, we test whether informing unaware consumers about the triggering event will affect results.

### Design

The actual boycott was directed against a ride-hailing company, which allegedly profited from a protest against President Trump’s executive order to ban refugees from certain countries from entering the United States. After the president's executive order, taxi drivers in New York City issued a public statement, refusing to pick up passengers at Kennedy Airport for one hour. Called upon to join this protest, the company refused. Instead, it posted a message on Twitter stating that for the duration of the taxi boycott surge pricing (an algorithm that raises the price of a ride during times of high demand) had been suspended for trips originating at JFK Airport. This behavior caused approximately 500,000 consumers to delete the app, effectively boycotting the provider. A hashtag encouraging deletion of the app spread rapidly and widely through social media at the heart of the protest against the company. As before, we highlighted that the reports are taken from real news websites.

In our study, 283 U.S. residents took part in an MTurk survey, with 220 participants completing all questions (*M*_age_ = 34.81, SD_age_ = 9.21; 75% male). MTurk randomly assigned participants to the study regardless of whether or not they were actual customers of the ridesharing company. Of our sample, 35 respondents had never used the ridesharing service and were therefore excluded, leaving a final sample of 185 participants for subsequent analyses. To increase validity, the questions assessing consumer knowledge of the case (65% had heard of the boycott) appeared at the beginning of the questionnaire such that respondents were not able to draw conclusions about study objectives. As with Study 3, respondents indicated their level of perceived egregiousness and boycott participation for both the time of the survey (*t*1 = twelve months after the boycott call) and, retrospectively, twelve months earlier (*t*0 = the time when the boycott was initiated).^8^

They also indicated whether or not they had actually known about the boycott. To those who did not, we presented a short newspaper article about the boycott. We re-employed Study 3's measures of boycott participation and perceived egregiousness. Measures of expressive and instrumental drivers were also identical to the ones used in Study 3. We used a dichotomous scale of subjective costs, asking respondents to affirm (1) or reject (0) the following statement: “I could not do without XY, because I do not have to wait for long until the driver picks me up.” All other scales consisted of seven-point Likert-type ratings. Table A2 in the Web Appendix holds scale means and statistics. Again, Fornell and Larcker’s (1981) criterion provides evidence for the discriminant validity of the constructs (Web Appendix Table A1). Results of the single-factor test (41.7%) indicated that common method variance did not bias the results. Inserting a theoretically uncorrelated marker item (“I like indie music”) also yields that the results are not distorted by a common method bias (Lindell & Whitney, 2001). Pearson's correlation coefficient indicates no significant correlation with any other item in the study.

### Results

Results of an initial t-test closely resemble those of previous studies, indicating that both perceived egregiousness (*M*_*t*0_ = 4.41, *M*_*t*1_ = 3.97, *t* = 5.62, *p* ≤ 0.001) and boycott participation (*M*_*t*0_ = 4.31, *M*_*t*1_ = 3.67, *t* = 5.79, *p* ≤ 0.001) decrease significantly from the heat-up phase (*t*0) to the cool-down phase (*t*1). Further results indicate that perceived egregiousness (*t*1) interacts significantly (*β* = 0.22, *t* = 2.03, *p* = 0.044) with boycott participation (*t*0) to affect boycotting at *t*1 (Table 6, Model 1). We next divided our sample into one group of participants who had known about the boycott and another group of participants who had first learned about the event during our study. With consumers who already knew about the event, the effects of promoters and inhibitors replicated the pattern found in previous studies (Table 6, Model 4). Furthermore, this study reveals an interaction of perceived egregiousness (*t*1) and service quality on boycott participation (Table 6, Model 4). In contrast with consumers who had not heard about the event, only two variables had a significant influence on boycott participation: self-enhancement (positively) and service quality (negatively) (Table 6, Model 3). This finding indicates that priming the story has no biasing effect. Testing interaction effects between “Boycott known = 1 (yes) vs. 0 (no)” and the other variables in our model indicates significant effects between prior knowledge of the boycott and perceived egregiousness, self-enhancement, brand image, perceived control, as well as service quality.

**Table 6:** Determinants of Boycott Participation (*t*1)—Study 4

	Model 1: Full Sample w. Interactions			Model 2: Full Sample wo. Interactions			Model 3: Not Known			Model 4: Known		
	*β*	*p*	*t*	*β*	*p*	*t*	*β*	*p*	*t*	*β*	*p*	*t*
*Controls*												
Age	− 0.01	0.433	− 0.79	0.00	0.573	− 0.56	− 0.09	0.163	− 1.41	0.04	0.328	0.99
Gender^a^	0.06	0.317	1.00	0.03	0.628	0.49	0.03	0.637	0.47	0.06	0.118	1.58
Income^b^	− 0.22	0.368	− 0.90	− 0.34	0.191	− 1.31	− 0.04	0.478	− 0.71	− 0.02	0.637	− 0.47
Education^c^	− 0.04	0.812	− 0.24	0.02	0.879	0.15	− 0.01	0.793	− 0.26	− 0.02	0.620	− 0.50
*Boycott and egregiousness*												
Boycott participation (*t*0)	0.13	0.376	0.89	0.08	0.083	1.75	0.03	0.593	0.54	0.35	0.000	3.87
Perceived egregiousness (*t*1)	− 0.08	0.719	− 0.36	0.42	0.000	3.92	0.30	0.003	3.09	0.10	0.134	1.52
Boycott participation (*t*0) × perceived egregiousness (*t*1)	0.22	0.044	2.03	0.24	0.006	2.79	− 0.01	0.873	− 0.16	0.16	0.020	2.38
*Boycott drivers*												
Self-enhancement	0.14	0.004	2.92	0.16	0.000	8.28	0.50	0.000	5.21	0.34	0.000	3.68
Brand image	− 0.53	0.012	− 2.54	− 0.07	0.289	− 1.06	0.11	0.142	1.48	− 0.15	0.005	− 2.90
Subjective costs	− 0.54	0.382	− 0.88	− 0.37	0.075	− 1.79	− 0.06	0.314	− 1.01	− 0.07	0.074	− 1.81
Perceived control	0.38	0.002	3.13	0.13	0.073	1.80	− 0.01	0.920	− 0.10	0.20	0.002	3.16
*Service*												
Service quality	0.11	0.314	1.01	− 0.05	0.180	− 1.35	− 0.24	0.006	− 2.85	0.00	0.240	0.07
Service quality × perceived egregiousness (*t*1)	0.29	0.140	1.48	0.16	0.036	2.12	− 0.02	0.735	− 0.34	0.16	0.006	2.30
*Story known interaction*												
Boycott participation (*t*0)	− 0.10	0.584	− 0.55									
Perceived egregiousness (*t*1)	0.40	0.004	2.94									
Boycott participation (*t*0) × perceived egregiousness (*t*1)	− 0.01	0.840	− 0.20									
Self-enhancement	− 0.01	0.957	− 0.05									
Brand image	0.38	0.015	2.46									
Subjective costs	0.14	0.731	0.34									
Perceived control	− 0.26	0.011	− 2.57									
Service quality	− 0.34	0.068	− 1.84									
Service quality × perceived egregiousness (*t*1)	− 0.01	0.357	− 0.79									
R^b^	0.84		0.87	0.87			0.89			0.87		
Adj. R^2^	0.84		0.84	0.84			0.87			0.75		
n	185			185			89			96		

Ordinary least squares regression

^a^Dummy coded: 0 = female; 1 = male

^b^Dummy coded: 0 = €1500 or less; 1 = €1501 or more

^c^Dummy-coded: 0 = high school diploma or lower; 1 = more than high school diploma

Yet again, we conducted between-subject t-tests to examine boycott drivers for the consumer types and respondents were assigned to one of the four groups. The *Deliberators* showed a significant increase in boycott participation (*M*_*t*0_ = 3.79, *M*_*t*1_ = 4.67, *t* = 7.00, *p* ≤ 0.001), while the boycotting level of the *Apathetic* remained constant (*M*_*t*0,*t*1_ = 3.87). The *Forgetters* displayed a decrease in boycott participation (*M*_*t*0_ = 3.83, *M*_*t*1_ = 3.14, *t* = 6.96, *p* ≤ 0.001) in combination with a decrease in perceived egregiousness (*M*_*t*0_ = 4.44, *M*_*t*1_ = 3.55, *t* = 13.10, *p* ≤ 0.001), whereas the *Capitulated* exhibited a decrease in boycott participation (*M*_*t*0_ = 4.46, *M*_*t*1_ = 3.93, *t* = 5.17, *p* ≤ 0.001) paired with an increase in perceived egregiousness (*M*_*t*0_ = 3.83, *M*_*t*1_ = 4.38, *t* = 4.73, *p* ≤ 0.001). Again, self-enhancement was a significant driver across groups (Table 3, lower panel). The results confirm that perceived control had a strong positive effect with the *Deliberators*. Remarkably, the *Apathetic's* behavior was driven by the inhibitor, subjective costs, rather than by the inhibitor, brand image. Perceived egregiousness was the key influencer for the *Forgetters*. For the *Capitulated* type, a loss in service quality was particularly relevant.

### Discussion

The findings obtained in Study 4 closely resemble the ones obtained in previous studies, suggesting that effects are stable and likely generalizable. Specifically, intrapersonal changes in perceived egregiousness and boycotting reemerged, as did the roles of instrumental and expressive promoters and inhibitors. Furthermore, Study 4 rules out the possibility that providing uninformed respondents with key facts of the egregious event biased the results.

## General Discussion

This paper conceptualizes and empirically tests intrapersonal changes in boycott participation. Four studies provide evidence for an integrative model consisting of a heat-up and a cool-down phase of boycotting. By providing a better understanding of the individual temporal dynamics of boycotting—especially intrapersonal changes—our research extends existing models that focus on the commencement of boycotts, thereby offering a unique contribution. In doing so, our study makes at least three important contributions.

**Fig. 3:**
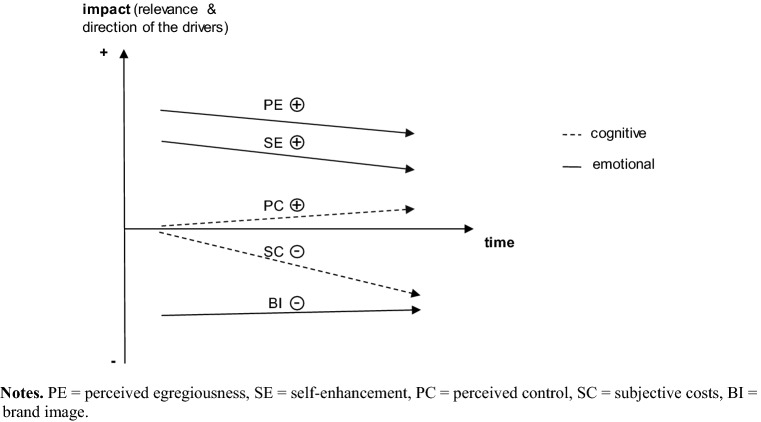
Dynamic nature of consumer boycotts. Notes. *PE* perceived egregiousness, *SE* self-enhancement, *PC* perceived control, *SC* subjective costs, *BI* brand image

First, we contribute to the boycott literature by detailing temporal changes in boycott participation at the individual consumer level. While previous studies establish an overall decline of consumers’ willingness to boycott over time, our findings, for the first time, illustrate the dynamic psychological aspects of boycotts at the individual level. We show that in the initial heat-up phase, boycott participation is primarily fueled by expressive drivers. During the following cool-down phase, additional instrumental drivers come into play, which, through more careful and rational consideration, can keep initial participants from further boycotting (Fig. 3).

This novel two-stage approach adds to the nascent literature on multi-stage models in sustainable consumption (Mai et al., 2021) and ties in not only with the hot/cold cognitions process explanation adopted for consumer response to ethical transgressions in sports contexts (e.g., Lee et al., 2016; Sato et al., 2018) but also with the “emotion-then-deliberation” sequence (Evans, 2008; Haidt, 2001). Our finding of diminishing importance of hot versus cold drivers is further also consistent with research on service failure recovery (e.g., Tsarenko & Tojib, 2011) and product-harm crisis (e.g., Khamitov et al., 2020). While Mai et al. (2021) put forward a similar multi-stage model in the context of organic consumption, ours is the first to offer a multi-stage view on why consumers start, sustain, and stop boycotting at different points in time. This temporal dynamic is especially evident in the empirical studies, where we cover time periods ranging from two weeks (Study 2) to 12 months (Study 4). Furthermore, validity is increased by assessing consumer reactions at two different points of time (Study 1 and Study 2), and by applying retrospective designs (Stud 3 and Study 4, Fig. 3).

Second, this novel dynamic psychological perspective enables us to extend existing boycotting models beyond a consumer’s initial decision to join by including the consumer's exit. Informed by research on the temporal effects of anger and revenge (e.g., Ettenson & Klein, 2005; Klein et al., 1998; Lee et al., 2016; Sato et al., 2018), this perspective adds to the research stream on consumer response to unethical firm behavior. Past research in this realm has predominantly focused on drivers to initially join boycotts (Hoffmann, 2011; Klein et al., 2004). We extend this literature by identifying and profiling four consumer types based on unique intrapersonal changes in boycotting behavior during the cool-down phase. Further to the four types, we disentangled the motives driving interpersonal differences between respondents with increasing levels of boycott participation and others with constant and decreasing levels. Finally, we show that the majority of consumers participate in a boycott to vent frustration.

Presenting a third contribution, we introduce two new service-related drivers of individual boycott participation that have not been studied previously. Since boycotting a company usually requires switching to another service provider, examining two novel service-related determinants provides more detail to subjective switching costs. Study 1's findings show that customer-friendly behavior of frontline employees can attenuate boycott participation over time. Findings obtained in Study 2 and Study 3 suggest that a strong customer-provider link and higher levels of perceived service quality can lower the likelihood of a boycott.

Finally, our four studies build confidence in the generalizability of findings, as the results appear robust across countries (Germany, U.S.), contexts (e-tailing, peer-to-peer ridesharing, video streaming, and hospitality), and study designs.

## Implications for Activists and Managers

### Strategic Considerations

Boycotts rarely maintain their level of intensity over time (Chavis & Leslie, 2009; Ettenson & Klein, 2005). However, many organizations that are targets of boycott calls still hope that the boycott has no sustainable impact on their behavior and that they are not required to change their policies. Many cases have, however, shown that a boycott can force a company to change its behavior (e.g., Shell’s Brent Spar crisis, Loefstedt and Renn 1997). In this line, our results demonstrate that there is a considerable share of consumers that do not just “cool down” after a while and revert to old consumption habits but rather stay angry or find rational arguments and measures to boycott the company. Companies targeted because of corporate ethical misbehaviors should therefore significantly adjust their activities or obligations to serve their internal and external stakeholder communities (*Corporate Social Responsibility (CSR)* Dahlsrud, 2008; Luo & Bhattacharya, 2006; Snider et al., 2003). Building on past research showing that corporate social responsibility (CSR) strategies can be advantegous for companies (Baron, 2001; McWilliams & Siegel, 2011) in terms of their brand equity (e.g., Torres et al., 2012; Yang and Basile 2020) or performance (e.g., Blasi et al., 2018; Luo & Bhattacharya, 2006), companies can interpret boycotts as a chance to adjust their CSR stategy instead of just awaiting the end of the boycott. Besides changing business practices and policies that have initially triggered a boycott, companies should reconsider the communication of their activities to avoid future negative consumer reactions that might induce boycott calls. Our results also show that frontline employees could be a good means for a company to interact with the customers. Other conditions that influence the impact of CSR activities should be considered as well, such as the fit between the company and the focal issue, the company reputation, the consumer-company identification, information transparency, and the physical environment (Bhattacharya & Sen, 2004; Kim, 2019; Kim & Kim, 2017; Lasarov et al., 2021; Wu et al., 2020). Besides these global CSR-related considerations, companies must develop strategies that are adjusted to the different consumer types we have identified in our research.

### Dynamic Considerations

Research on crises management has identified a number of situations where consumers were not satisfied with corporate behaviors, such as actions deemed unethical (Coombs, 2004, 2007; Coombs & Holladay, 2002; Huang, 2006). According to the results, an organization’s crises managers should first assess the degree of “guilt” attributable to the organization and then develop appropriate response strategies. According to our results, there are four strategies that might help mitigate consumer boycotts. *First,* maintaining a positive company image would be an effective means of buffering against or at least attenuating the negative consequences of boycotts. Past research has identified a number of measures for enhancing brand image and behavioral loyalty; in the context of social boycotts, CSR activities (Aguinis & Glavas, 2012; Ailawadi et al., 2014; Barnett et al., 2020) and cause-related marketing (Chen & Huang, 2016) might mostly impact the company’s brand image. However, since our results show that also aspects not related to the company’s CSR measures (e.g., the company delivers its products at the promised time) can boost the company image, boycott activists could counter these measures that are not related to CSR by continuously running campaigns pointing out the boycotted company's ethical wrongdoings. Boycott activists may therefore work to maintain high levels of negative emotions against the boycotted company. *Second,* our results indicate that high switching costs incurred, for example, through the search for alternative products or services, can serve as a buffer against sustained boycotting. Boycott activits may therefore provide customers with information about potential substitutes (e.g., other retailers, producers, servide providers, products) to decrease switching costs and to ease the switch to competitors. On the other hand, to reduce the likelihood of extended boycotting, managers should consider raising boycott-related barriers, such as the (non-)monetary value of seeking alternatives. *Third*, we found that a high service quality can serve as an effective buffer against calls for boycotting. Companies should, therefore, maintain sufficiently high levels of service quality as a means to communicate more directly and competently with their customers. Similarly, activists should be aware of the high importance of service quality; to counter it, they could emphasize better service of a competitor as a motivation to switch. *Fourth,* in many cases frontline employees are the main touch points linking the company with its customers (Hartline et al., 2000) and, therefore, the frontline employees are most likely to gather first-hand customer insights (Coelho et al., 2011). Since frontline employees are positioned best to listen to and understand customers, we suggest that frontline employees should spearhead companys' efforts to deal with boycotts. Their key task would be to identify the customer types involved (i.e., the Capitulated), and then implement the type-specific measures outlined above.

### Different Consumer Types

Furthermore, our study distinguishes different consumer types that have individual boycott dynamics, which boycott activists and managers must consider. In this regard, only a few consumers eventually stop boycotting or do not start boycotting at all (Apathetics, Forgetters), while others maintain higher levels of perceived egregiousness over time and stop boycotting only because of individual cost–benefit considerations (the Capitulated). Yet, another consumer type starts boycotting only after a certain amount of time has passed (the Deliberators). Our findings can help boycott activists increase the success of their boycott in the long term. On the other hand, companies could use our findings to soften the negative boycott dynamics for avoiding damage in the short term. In response to the boycott call, the company might address the cause and remedy its unethical behavior that initially triggered the boycotts. Table 7 holds more detailed advice for both activists and managers on how to more specifically address each of the four consumer types identified in our research.

**Table 7:** Characteristics, Conclusions, and Implications by Consumer Type

Consumer type	Temporal Effects		Conclusion	Implications	
	PE	BOY		For activists	For managers
The apathetic	→ →		The boycott decision of the Apathetic is driven by a positive evaluation of the target company. They have initially decided not to boycott the firm, or they keep boycott participation constantly on a low level	Boycott activists and nongovernmental organizations should keep emphasizing the misbehavior of the company to convince loyal consumers to reconsider their perception of the brand. However, certain of those consumers will still not participate in the boycott →Expressively driven (triggering emotions)	To prevent consumers from participating in boycotts and reacting apathetically to transgressions, managers should build up a strong image of the company. Moreover, the company should encourage the Apathetic to convince other customers to stay with the company →Expressively driven (strengthen brand image)
The forgetters	↘↘		The Forgetters initially support the boycott. Yet, after a certain amount of time has elapsed and the media has stopped reporting, the Forgetters revert to old consumption habits	Since the Forgetters are mainly driven by emotional components (brand image, perceived egregiousness), activists should approach this segment with emotional appeals. They should constantly draw the attention of these consumers to the misbehavior of the boycotted company to keep levels of egregiousness high → Expressively driven (triggering emotions)	To deal with the Forgetters, managers should wait until the media coverage of the event depletes and the consumers' egregiousness decreases. CSR activities, cause-related marketing, and changing the company policies in response to the boycott call (e.g., raising working conditions of employees) can therefore help loweing negative public attention →Expressively driven (awaiting the cool-down phase)
The capitulated	→ ↘		The Capitulated emphasize boycott-related costs and wish to boycott, but they recognize that obstacles are higher than personal rewards. They therefore stop participating in the boycott	Activists should communicate measures to minimize switching costs. For example, they could provide informtation on competitors with similar products and services. Since these substitutes could be more expensive, activists could emphasize other reasons (e.g., product quality, service quality) to justify the switching costs →Instrumentally driven (supporting information to minimize subjective costs)	Managers should consider boycott-related costs, such as consumers’ financial efforts for seeking alternatives. They may therefore increase behavioral loyalty by increasing switching costs for consumers. For example, companies may decrease the prices for loyal consumers (e.g., by offering discounts for products) → Instrumentally driven (emphasizing boycott-related barriers)
The deliberators	→ ↗		The Deliberators initially do not participate in a boycott. After observing the consequences of the boycott and reconsidering the perceived control, they join the boycott at a later stage in time	Activists should continously inform the public about the consequences of the boycott and demonstrate the impact of the boycotters (e.g., changing behavior of the company). Furthermore, the instrumental goals of the boycott call should be communicated to emphasize the instrumental elements of the boycott → Instrumentally driven (supporting information about the impact)	In most cases, consumers’ actions do not extend sustainable impact on corporate behavior. Managers should, however, consider the possible temporal effects that could evolve after an egregious act and implement crises management measures to react on boycott calls. As for the Forgetters type, significant changes of the company policies in response to the boycott call might help → Instrumentally driven (situational crisis management)

PE = Perceived Eregiousnes, BOY = Boycott Participation; → constant levels over time, **↘** decreasing levels over time, ↗ increasing levels over time

## Limitations and Further Research

This study has a few limitations, offering opportunities for further research. Conceptually, we make use of established theories to put forward the two-stage model of emotional heating and cognitive cooling. We caution readers, however, that a hard distinction between the two stages may be misleading and a more nuanced view may be useful for obtaining further insights. Methodologically, our measurement of boycott participation is based on self-reported scales and past behavior. While we validated these measures with a behavioral variable (lottery) in Study 2 and Study 3 to ensure the robustness of our finding, employing more behavioral variables for researching the temporal dynamics of boycotting may provide additional insights.

A few other avenues for future research need mentioning. From a conceptual perspective, our research is based on a cost–benefit model (Klein et al., 2004) accounting for instrumental and expressive determinants. Presenting an alternative, behavioral models (e.g., Hahn and Albert (2017)) introduce the notion of strong reciprocity to the boycott literature and analytically separate two behavioral models: a self-regarding type (= driven by the maximization of private utility) and a strongly reciprocal type (= driven by a desire to reciprocate the (un)fair behavior of others). Adapted to our context, strongly reciprocal consumers might initially perceive higher levels of egregiousness and might be more willing to maintain their boycott even when the strategic conditions turn to be (more) unfavorable in the long run (e.g., increasing switching costs). Future research may thus find it valuable to categorize consumer types based on behavioral models and examine subsequent differences in boycott dynamics. An initial attempt could be to categorize determinants according to the proposed categories: According to Hahn and Albert (2017), self-regarding people are motivated by the maximization of their private utility, and they weigh expected private costs against expected private benefits for different alternatives and use that information to make choices. On the other hand, strongly reciprocal actors are willing to sanction the perceived (un)fairness of others, using punishments (negative sanctions) or rewards (positive sanctions), even if doing so decreases their payoffs and entails additional net costs for them (Hahn & Albert, 2017). Our research may integrate well with the conceptualization of Hahn and Albert (2017) for a number of reasons: Specifically, strongly reciprocal consumers are more likely than self-regarding consumers to boycott the target firm, even when the likelihood of a change of the firm’s behavior is low. In this respect, these consumers should have higher levels of perceived control (an instrumental factor) and, therefore, keep boycotting.

Second, researchers may find it beneficial to examine the role of social media (Graf-Vlachy et al., 2020) in the development and temporal dynamics of a boycott. A post hoc analysis of Study 4's data shows that of those respondents who had previously heard about the unethical incident, 85.6% had received this information through social media (48.3% via Facebook, 40.7% via Twitter, 14.4% via Instagram, and 20.3% via other social media; multiple choice possible). In contrast, only 16.1% had read about the incident in a newspaper, and 17.8% had learned about it via TV. Researchers may thus find it beneficial to relate media usage to our boycotter types and disentangle temporal dynamics by primary sources of media used.

Third, going beyond consumer reactions to corporate misconduct, other stakeholders (e.g., the employees) should be examined. Prior research indicates that stakeholder attention after a company’s misconduct varies, often failing to result in retribution (Barnett, 2014). This is important, as the firm’s exposure to stakeholders may have a relatively stronger impact on managerial decision making than economic performance or the degree of CSR exhibited (Chiu & Sharfman, 2011).

Fourth, our study did not fully explore the emotional costs assosciated with boycotts. For example, future studies could examine the influence of related concepts, such as brand attachment (Malär et al., 2011) or brand love (Batra et al., 2012). In line with the stereotype content model (Fiske et al., 2002) and its two fundamental dimensions of social perception (warmth and competence), this research focused on quality and competence-related aspects that prevent consumers from boycotting, for example, service quality, quality of products, and service of frontline employees. Future research may find it worthwhile to examine the difference between competence-related costs (e.g., quality, trust) and warmth-related aspects (e.g., communication style of the frontline employees).

Last, more work is needed to better integrate other domains that are intrinsically related to ethical issues. For example, research on consumer animosity suggests that consumers tend to express their anger toward a nation by boycotting the nation's products and brands (Ettenson & Klein, 2005; Hoffmann et al., 2011; Klein et al., 1998). In a recent example, speculations about the origin of the COVID-19 pandemic caused many consumers and even the U.S. government to blame China for its misbehavior during the first outbreak and caused boycott calls toward Chinese products (Sevastoulo & Manon, 2020; Krueger et al. 2020). Researching the temporal dynamics of country-related boycotts may thus improve understanding of boycott dynamics in an animosity context.

## Supplementary Information

Below is the link to the electronic supplementary material.

Supplementary file1 (DOCX 194 kb)

## Footnotes

1 Friedman’s (2002) taxonomy of boycotts on the macro level also includes punitive and catalytic boycotts. These are, however, less frequent and relevant for the present consideration of intrapersonal variations in boycott motivations on the micro level.

2 To obtain a rough estimation of the duration of the heat-up phase, we analyzed the media coverage of an event that was a trigger for a boycott call as a proxy for the consumer egregiousness. The results show a sharp decrease of coverage after two weeks. The time lags in all empirical studies therefore encompass at least two weeks. The study description and results are detailed in the Appendix (Pretest of Study 3).

3 We conducted t-tests for two independent samples to test whether the items that were included for the respondents who participated in both rounds differed from all the items that were included for those who participated in the first round only. Fifty-six percent of the participants who participated in the first round were also surveyed in the second round. The results show that there is no bias, because all the t-tests show no significant effects.

4 Of all respondents filling in the first survey, 37% also participated in the second survey. T-tests for two independent samples were run to establish if all the items that were included for the respondents who participated in both rounds differed from all the items that were included for those who participated in the first round only. None of the t-tests yielded significant results, showing that there is no bias.

5 As a robustness check, we conducted regression analyses for the boycott intention (“I was sure that I would avoid buying products on XY in the future”) and self-reported boycott participation (“Immediately after learning about the event, I consciously restrained my consumer behavior toward XY”) separately as dependent variables. The results show no difference to the regression analyses in which we used the boycott participation construct. Regression coefficients for boycott intention (*t*1) as dependent variable: boycott intention (*t*0): .13***, perceived egregiousness (*t*1): .18***, boycott intention (*t*0) × perceived egregiousness(*t*1): .38***, self-enhancement: .15*, brand image: -.19***, perceived control: .09^+^, subjective costs: -.11*. Regression coefficients for self-reported boycott behavior (*t*1) as dependent variable: self-reported boycott behavior (*t*0): .08*, perceived egregiousness (*t*1): .20***, self-reported boycott behavior (*t*0) × perceived egregiousness (*t*1): .46***, self-enhancement: .13*, brand image: -.18***, perceived control: .08^+^, subjective costs: -.12*.

6 Based on Podsakoff et al.'s (2003) critical discussion regarding the validity on this method, Fuller et al. (2016) ran a Monte Carlo simulation that show the test can indeed detect biasing levels of CMV, mostly under conditions that are common in survey-based marketing research.

7 Echambadi and Hess (2007) showed that the VIF in moderated multiple regression models underestimate the multi-collinearity problem if the indicators were centered. We therefore reran the analysis with the indicators without centering. Still, the maximum VIF ≤ 3.07. Accordingly, there is no severe distortion due to multi-collinearity.

8 Other than in Study 3, we replaced one of the three items that measured perceived egregiousness. We included "After learning about this event, I thought XY’s handling in this situation was socially acceptable" in Study 4 instead of "After learning about this event, I thought XY’s handling of their employees was socially acceptable," as the egregious behavior in Study 3 was related to the situation of the employees, whereas in Study 4 it was related to the companies' exploitation of a political boycott.
