# Supplementary material for: Vanishing Boycott Impetus: Why and How Consumer Participation in a Boycott Decreases Over Time
Source: J Bus Ethics. 2021 Nov 24;182(4):1129–54. doi: 10.1007/s10551-021-04997-9 (PMC8612116; doi:10.1007/s10551-021-04997-9)
Supplement: Supplementary file 1 — Supplementary file1 (DOCX 194 kb) [file 10551_2021_4997_MOESM1_ESM.docx]

# APPENDICES

# TABLE A1

**Correlation Matrix**

**Study 1**


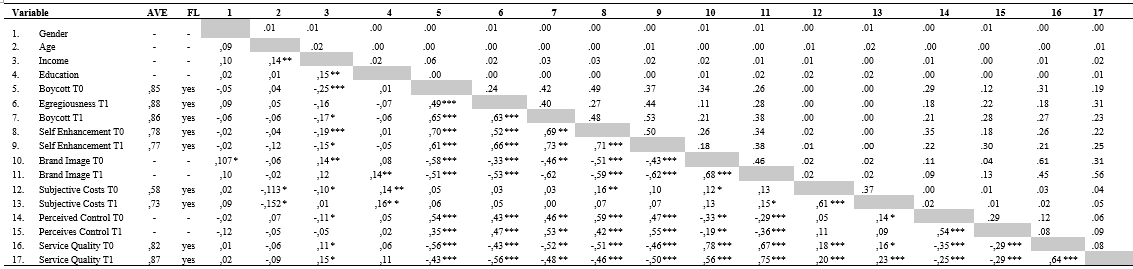


Notes: Pearson's product moment correlation. Level of significance: * *p* ≤ .05, ** *p* ≤ .01, **** p* ≤ .001. AVE=Average variance extracted. F/L=Fornell/Larcker criterion. Table shows correlation coefficients (left of the main diagonal) and squared correlation coefficients (right of the main diagonal).

**Study 2**

Notes: Pearson's product moment correlation. Level of significance: * *p* ≤ .05, ** *p* ≤ .01, **** p* ≤ .001. AVE=Average variance extracted. F/L=Fornell/Larcker criterion. Table shows correlation coefficients (left of the main diagonal) and squared correlation coefficients (right of the main diagonal).

**Study 3**

|  | | **AVE** | **F/L** |  | **1** |  | **2** |  | **3** |  | **4** |  | **5** |  | **6** |  | **7** | **8** | |  | **9** |  | **10** |  | **11** |  |
| --- | --- | --- | --- | --- | --- | --- | --- | --- | --- | --- | --- | --- | --- | --- | --- | --- | --- | --- | --- | --- | --- | --- | --- | --- | --- | --- |
| **1** | Gender | **-** | **-** |  |  |  | .00 |  | .00 |  | .00 |  | .06 |  | .00 |  | .01 |  | .06 |  | .01 |  | .01 |  | .00 |  |
| **2** | Age | **-** | **-** |  | .06 |  |  |  | .01 |  | .01 |  | .00 |  | .00 |  | .00 |  | .00 |  | .02 |  | .01 |  | .00 |  |
| **3** | Income | **-** | **-** |  | .00 |  | .10 |  |  |  | .01 |  | .01 |  | .00 |  | .01 |  | .00 |  | .00 |  | .00 |  | .00 |  |
| **4** | Education | **-** | **-** |  | -.01 |  | .08 |  | .11 |  |  |  | .00 |  | .01 |  | .00 |  | .00 |  | .00 |  | .00 |  | .00 |  |
| **5** | Boycott participation t0 | .81 | yes |  | -.24 | ** | .03 |  | .09 |  | -.01 |  |  |  | .10 |  | .52 |  | .64 |  | .12 |  | .00 |  | .18 |  |
| **6** | Perceived egregiousness t1 | .64 | yes |  | -.06 |  | .01 |  | -.02 |  | -.08 |  | .32 | *** |  |  | .28 |  | .15 |  | .04 |  | .00 |  | .05 |  |
| **7** | Boycott participation t1 | .78 | yes |  | -.11 |  | .03 |  | .09 |  | -.06 |  | .72 | *** | .53 | *** |  |  | .49 |  | .18 |  | .01 |  | .15 |  |
| **8** | Self-enhancement | .65 | yes |  | -.24 | *** | -.01 |  | .04 |  | -.05 |  | .80 | *** | .38 | *** | .70 | *** |  |  | .10 |  | .01 |  | .19 |  |
| **9** | Brand image | .84 | yes |  | .11 |  | -.13 | * | -.03 |  | .00 |  | -.34 | *** | -.20 | *** | -.43 | *** | -.31 | *** |  |  | .03 |  | .01 |  |
| **10** | Subjective costs | .92 | yes |  | -.12 |  | -.10 |  | -.03 |  | .03 |  | .04 |  | .01 |  | -.09 |  | .08 |  | .18 | ** |  |  | .00 |  |
| **11** | Perceived control | .84 | yes |  | .00 |  | .04 |  | .03 |  | -.07 |  | .42 | *** | .22 | *** | .39 | *** | .43 | *** | -.08 |  | .05 |  |  |  |

Notes: Pearson's product moment correlation. Level of significance: * *p* ≤ .05, ** *p* ≤ .01, *** *p* ≤ .001. AVE=Average variance extracted. F/L=Fornell/Larcker criterion. Table shows correlation coefficients (left of the main diagonal) and squared correlation coefficients (right of the main diagonal).

**Study 4**

|  | | **AVE** | **F/L** |  | **1** |  | **2** |  | **3** |  | **4** | |  | | **5** | |  | | **6** | |  | | **7** | | **8** | | | |  | | **9** | |  | | **10** | |  | | **11** | |  | | **12** | |  | |  |
| --- | --- | --- | --- | --- | --- | --- | --- | --- | --- | --- | --- | --- | --- | --- | --- | --- | --- | --- | --- | --- | --- | --- | --- | --- | --- | --- | --- | --- | --- | --- | --- | --- | --- | --- | --- | --- | --- | --- | --- | --- | --- | --- | --- | --- | --- | --- | --- |
| **1** | Gender | - | - |  |  |  | .07 |  | .00 |  | .00 | |  | | .01 | |  | | .00 | |  | | .00 | |  | | .01 | |  | | .00 | |  | | .00 | |  | | .00 | |  | | .00 | |  | |  |
| **2** | Age | - | - |  | .26 | *** |  |  | .00 |  | .00 | |  | | .00 | |  | | .00 | |  | | .01 | |  | | .02 | |  | | .00 | |  | | .00 | |  | | .01 | |  | | .00 | |  | |  |
| **3** | Income | - | - |  | -.01 |  | .04 |  |  |  | .00 | |  | | .01 | |  | | .04 | |  | | .01 | |  | | .03 | |  | | .00 | |  | | .00 | |  | | .02 | |  | | .02 | |  | |  |
| **4** | Education | - | - |  | .04 |  | .01 |  | .03 |  |  | |  | | .00 | |  | | .00 | |  | | .00 | |  | | .00 | |  | | .02 | |  | | .01 | |  | | .00 | |  | | .01 | |  | |  |
| **5** | Boycott participation t0 | .79 | yes |  | -.08 |  | -.03 |  | .08 |  | .04 | |  | |  | |  | | .52 | |  | | .59 | |  | | .66 | |  | | .04 | |  | | .02 | |  | | .40 | |  | | .11 | |  | |  |
| **6** | Perceived egregiousness t1 | .90 | yes |  | -.05 |  | -.07 |  | .21 | ** | | .03 | |  | | .72 | | *** | |  | |  | | .58 | |  | | .61 | |  | | .05 | |  | | .01 | |  | | .48 | |  | | .26 | |  | |
| **7** | Boycott participation t1 | .86 | yes |  | -.05 |  | -.09 |  | .11 |  | .03 | |  | | .77 | | *** | | .76 | | ** | |  | |  | | .74 | |  | | .07 | |  | | .05 | |  | | .54 | |  | | .18 | |  | |  |
| **8** | Self-enhancement | .73 | yes |  | -.10 |  | -.13 |  | .17 | * | .07 | |  | | .81 | | *** | | .78 | | ** | | .86 | | *** | |  | |  | | .03 | |  | | .01 | |  | | .63 | |  | | .11 | |  | |  |
| **9** | Brand image | - | - |  | -.05 |  | -.01 |  | .00 |  | .13 | |  | | -.21 | | ** | | -.23 | | *** | | -.27 | | *** | | -.18 | | ** | |  | |  | | .02 | |  | | .01 | |  | | .35 | |  | |  |
| **10** | Subjective costs | - | - |  | .02 |  | .02 |  | .02 |  | .10 | |  | | -.13 | |  | | -.11 | |  | | -.22 | | ** | | -.11 | |  | | .13 | | * | |  | |  | | .02 | |  | | .03 | |  | |  |
| **11** | Perceived control | .88 | yes |  | -.05 |  | -.08 |  | .12 |  | .07 | |  | | .63 | | *** | | .69 | | *** | | .74 | | *** | | .80 | | ** | | -.08 | |  | | -.13 | |  | |  | |  | | .12 | |  | |  |
| **12** | Service quality | - | - |  | .00 |  | -.02 |  | -.15 | * | .08 | |  | | -.33 | | *** | | -.51 | | *** | | -.42 | | *** | | -.34 | | *** | | .59 | | *** | | .17 | | * | | -.34 | | *** | |  | |  | |  |

Notes: Pearson's product moment correlation. Level of significance: * *p* ≤ .05, ** *p* ≤ .01, *** *p* ≤ .001. AVE=Average variance extracted. F/L=Fornell/Larcker criterion. Table shows correlation coefficients (left of the main diagonal) and squared correlation coefficients (right of the main diagonal).

**TABLE A2
Measurement Scales**

| **Scales / Items** | | **Studies** | **Study 1** | | |  | **Study 2** | | |  | **Study 3** | | |  | **Study 4** | | |
| --- | --- | --- | --- | --- | --- | --- | --- | --- | --- | --- | --- | --- | --- | --- | --- | --- | --- |
|  | |  | *α* | *M* | *SD* |  | *α* | *M* | *SD* |  | *α* | *M* | *SD* |  | *α* | *M* | *SD* |
| *Boycott participation (t0)^1^* | |  | .83 | 4.32 | 1.67 |  | .86 | 3.31 | 1.83 |  | .88 | 3.36 | 1.58 |  | .90 | 4.31 | 1.94 |
|  | I restrain my consumption habits on XY. | 1,2,3,4 |  |  |  |  |  |  |  |  |  | | |  |  | | |
|  | One should participate in boycotting XY. | 1,2,3,4 |  |  |  |  |  |  |  |  |  |  |  |  |  |  |  |
|  | I am sure that I won't buy products from XY  anymore. | 3,4 |  |  |  |  |  |  |  |  |  |  |  |  |  |  |  |
|  | I will boycott XY. | 2 |  |  |  |  |  |  |  |  |  |  |  |  |  | | |
| *Boycott participation (t1)* | |  | .77 | 3.66 | 1.65 |  | .86 | 3.08 | 1.76 |  | .84 | 2.82 | 1.36 |  | .92 | 3.67 | 1.80 |
|  | I restrain my consumption habits on XY. | 1,2,3,4 |  |  |  |  |  |  |  |  |  |  |  |  |  |  |  |
|  | One should participate in boycotting XY. | 1,2,3,4 |  |  |  |  |  |  |  |  |  |  |  |  |  |  |  |
|  | I am sure that I won't buy products from XY  anymore. | 3,4 |  |  |  |  |  |  |  |  |  | | |  |  | | |
|  | I will boycott XY. | 2 |  |  |  |  |  |  |  |  |  |  |  |  |  | | |
| *Perceived egregiousness (t0)^1^* | |  | .83 | 4.51 | 1.51 |  | .85 | 3.64 | 1.63 |  | .87 | 5.90 | .70 |  | .78 | 4.41 | 1.38 |
|  | I do not agree with XY's business practice. | 1,2,3,4 |  |  |  |  |  |  |  |  |  |  |  |  |  |  |  |
|  | When thinking of XY's business practice, I feel angry. | 1,2,3,4 |  |  |  |  |  |  |  |  |  |  |  |  |  |  |  |
|  | XY's handling of their employees is socially acceptable. | 3,4 |  |  |  |  |  |  |  |  |  | | |  |  |  |  |
| *Perceived egregiousness (t1)* | |  | .84 | 3.68 | 1.51 |  | .88 | 3.37 | 1.61 |  | .89 | 5.14 | .92 |  | .77 | 3.97 | 1.46 |
|  | I do not agree with XY's business practice. | 1,2,3,4 |  |  |  |  |  |  |  |  |  |  |  |  |  |  |  |
|  | When thinking of XY's business practice, I feel angry. | 1,2,3,4 |  |  |  |  |  |  |  |  |  |  |  |  |  |  |  |
|  | XY's handling of their employees is socially acceptable. | 3,4 |  |  |  |  |  |  |  |  |  | | |  |  |  |  |
| *Perceived control^2^* | |  | - | 3.71 | 1.75 |  | - | 3.65 | 1.75 |  | .81 | 4.19 | 1.49 |  | .75 | 4.27 | 1.50 |
|  | By boycotting products of XY, I can contribute to a shift of the management's behavior. | 1,2,3,4 |  |  |  |  |  |  |  |  |  |  |  |  |  |  |  |
|  | Boycotting is an appropriate means to discourage corporations | 3,4 |  |  |  |  |  |  |  |  |  |  |  |  |  |  |  |
|  | from taking actions that harm society. | 3,4 |  |  |  |  |  |  |  |  |  |  |  |  |  |  |  |
| *Self-enhancement (exit) ^2^* | |  | .88 | 3.60 | 1.56 |  | .90 | 3.07 | 1.63 |  | .89 | 3.23 | 1.49 |  | .94 | 3.54 | 1.56 |
|  | I would feel guilty if I kept on purchasing products from XY. | 1,2,3,4 |  |  |  |  |  |  |  |  |  |  |  |  |  |  |  |
|  | I would feel ashamed if people boycotting XY saw me still using the services of XY. | 1,2,3,4 |  |  |  |  |  |  |  |  |  |  |  |  |  |  |  |
|  | My friends and family support me in not buying products from XY anymore. | 1,2,3,4 |  |  |  |  |  |  |  |  |  |  |  |  |  |  |  |
|  | If I boycott XY, I am at peace with myself. | 3,4 |  |  |  |  |  |  |  |  |  |  |  |  |  |  |  |
|  | I think there is a moral obligation to show one's solidarity with the employees of XY by boycotting their products. | 3,4 |  |  |  |  |  |  |  |  |  |  |  |  |  |  |  |
| *Self-enhancement (voice) ^2^* | |  | .90 | 4.06 | 1.36 |  |  |  |  |  |  |  |  |  |  |  |  |
|  | Sharing my experience at the restaurant will help me create a positive self-impression among others. | 1 |  |  |  |  |  |  |  |  |  |  |  |  |  |  |  |
|  | Sharing my experience at the restaurant will help me receive positive feedback from others. | 1 |  |  |  |  |  |  |  |  |  |  |  |  |  |  |  |
|  | Sharing my experience at the restaurant will help me create the impression of me as a good person. | 1 |  |  |  |  |  |  |  |  |  |  |  |  |  |  |  |
| *Brand image^2^* | |  | - | 5.03 | 1.54 |  | - | 5.78 | 1.18 |  | .80 | 5.88 | 1.06 |  | .66 | 4.99 | 1.20 |
|  | XY delivers its products at the promised time. | 3 |  |  |  |  |  |  |  |  |  |  |  |  |  |  |  |
|  | I feel safe when using XY's services. | 3,4 |  |  |  |  |  |  |  |  |  |  |  |  |  |  |  |
|  | I like XY. | 1,2 |  |  |  |  |  |  |  |  |  |  |  |  |  |  |  |
| *Subjective costs^2^* | |  | - | 3.27 | 1.46 |  | - | 5.22 | 1.77 |  | .91 | 4.12 | 1.64 |  | - | .11 | .32 |
|  | It is very costly to look for an alternative to XY. | 1,2,3 |  |  |  |  |  |  |  |  |  |  |  |  |  |  |  |
|  | I could not do without XY because of the variety of movies, series, and programs they offer. | 2 |  |  |  |  |  |  |  |  |  |  |  |  |  |  |  |
|  | It takes a lot of time to look for a different fast food restaurant (*study 1*) / video streaming service (*study 2*) / e-retailer (*study 3*) such as XY. | 2,3 |  |  |  |  |  |  |  |  |  |  |  |  |  |  |  |
|  | There is no appropriate alternative to XY. | 2,3 |  |  |  |  |  |  |  |  |  |  |  |  |  |  |  |
|  | I could not do without XY, because I do not have to wait for a long time till the driver picks me up (*binary*). | 4 |  |  |  |  |  |  |  |  |  |  |  |  |  |  |  |
| *Perceived service quality^2^* | |  | .87 | 4.20 | 1.52 |  | .84 | 5.42 | .63 |  |  |  |  |  | .87 | 4.51 | 2.77 |
|  | Overall, XY’s customer service is excellent. | 1,2,3 |  |  |  |  |  |  |  |  |  |  |  |  |  |  |  |
|  | I am very satisfied with XY. | 1,2,3 |  |  |  |  |  |  |  |  |  |  |  |  |  |  |  |
| *Service of frontline employees^2^* | |  | - | 4.65 | 1.44 |  |  |  |  |  |  |  |  |  |  |  |  |
|  | The service of XY’s frontline employees is excellent. | 1 |  |  |  |  |  |  |  |  |  |  |  |  |  |  |  |

Notes: α = Cronbach’s alpha; M=Mean value; SD=Standard deviation; ^1^ in Study 3 and Study 4 the questions were formulated in the past tense, as we retrospectively asked participants (e.g., "after hearing about this event, one should have participated in boycotting XY" (Boycott), or "after learning about this event, I didn't agree with XY’s business practice" (Perceived egregiousness)). ^2^  In Study 1 and Study 2 the promoters, inhibitors, and service-specific items were measured at both points of time (t0, t1). For the sake of clearity, the table only shows the α, M, and SD values for t1. Values for t0 are similar to the presented values and can be provided upon request.

# Appendix A3 – Vignettes

| **Study 1: McDonalds** |
| --- |
| Recently, McDonalds repeatedly made the news through media reports of questionable business practices. For example, in February 2019 McDonalds was a target of the #RaiseTheWage-Campaign. This happened after an American politician serving as the U.S. Representative for Minnesota's fifth congressional district called attention to the following: In 2017, Steve Easterbrook, the CEO of McDonalds, received a salary of $21.8 million, whereas the average McDonalds worker was paid only $7.00 per hour. A second news story centered on a billboard put up on New York’s Times Square on New Year’s Eve. Laconically stating “New Year. Same Cruelty,” the billboard called attention to the suffering of chickens on the McDonalds farms. The web page for the campaign behind the billboard, “truthaboutmcdonalds.com,” rapidly attracted millions of visitors when it was launched in 2018. In a third media case, McDonalds workers, encouraged by the #MeToo movement, elected to stage a one-day strike at restaurants in ten major cities, hoping to push management to take stronger action against on-the-job sexual harassment in the firm. In light of these events, customers of McDonalds have started considering to boycott the company and to switch to other fast food providers. In this survey, we are interested in how you feel about McDonalds, specifically your intention to boycott. |
| *Sources:*  <https://www.politifact.com/factchecks/2019/feb/12/ilhan-omar/omar-trips-details-mcdonalds-ceo-and-typical-worke/>  <https://www.livekindly.co/boycott-mcdonalds-billboard-times-square/>  <https://www.theguardian.com/business/2018/sep/12/mcdonalds-workers-set-to-strike-over-sexual-harassment> |

| **Study 2: Netflix** |
| --- |
| Recently, Netflix repeatedly made the news in reports alleging questionable business practices and methods. For example, Netflix’s new reality show, *The Push*, was accused of manipulating unknowing participants to commit murder. In the show, psychological illusionist Derren Brown maliciously places an unsuspecting participant in a situation where committing murder appears to be the only way out. A group of actors conspires to trick the candidate into actually participating in the killing of a human being (at least that’s how the candidate sees it). In a second case, a Florida woman blames Netflix for her daughter’s recent attempt at suicide. She claims that it was viewing Netflix’s *13 Reasons Why* that caused her daughter to reenact the main character’s death after being egged on by her friends. In fact, according to a study published in the *American Journal of Medicine*, a key scene in *13 Reasons Why* is being blamed for a significant rise in suicide rates. According to the study’s authors, within three weeks of the series’ launch, internet searches for the key word “suicide” rose by 19%. In a third case, Netflix comedian Daniel Sloss brags that the jokes in his show caused more than 4,500 relationships being broken up, 16 engagements cancelled, and 20 couples divorced. In light of these news, Netflix customers have started considering to boycott the company and to switch to other providers. In this survey, we are interested in your reactions, specifically your intention to boycott Netflix. |
| *Sources:*  <https://www.thesun.co.uk/news/5641793/this-new-netflix-show-starring-derren-brown-wants-to-manipulate-people-into-committing-murder/>  <https://www.foxnews.com/entertainment/13-reasons-why-slammed-by-florida-mom-following-teen-daughters-suicide-attempt>  <https://www.youtube.com/watch?v=aj0Y6NtsSiM> |

| **Study 3: Amazon** |
| --- |
| At the beginning of February, the ARD showed the documentary *Ausgeliefert! Leiharbeit bei Amazon*. The documentary shows how Amazon recruits thousands of workers as auxiliary staff (3,300; 3,100 of them temporary) from all over Europe for the upcoming Christmas business. The documentary showcases how the staff is lured with false information about their salary. Furthermore, the employees are housed in vacation villages where they live in shared accomodations with up to six other employees. Finally, the staff is monitored by a security service that massively intimidates the employees and that has full-time access to the workers' accommodations.  Moreover, the security company was said to have connections to the right-wing extremist scene as evidenced by the clothing of the security men shown. Finally, it is known that Amazon increasingly relies on temporary workers. The documentary shows the interaction between Amazon, employment agencies, temporary employment agencies, and transport companies, and everything that goes with it: the cramped conditions in which the temporary workers live. The documentary also shows the overcrowded buses that run only once per shift—if the workers are late, they will receive even less pay. |
| *Sources:*  Spiegel.de (13.02.2013). ARD Reportage dokumentiert Missstaende in der Leiharbeit bei Amazon, http://www.spiegel.de/wirtschaft/unternehmen/ard-reportage-dokumentiert-missstaende-in-der-leiharbeit-bei-amazon-a-883156.html, gesehen am 19.09.13. |

| **Study 4: Uber** |
| --- |
| #DeleteUber In 2017, there was a boycott against the ride-hailing service, Uber, because it intended to profit from a protest against President Trump’s executive order banning refugees and immigrants from certain countries from entering the United States. After the president issued his executive order, taxi drivers in New York City issued a statement refusing to pick up passengers at Kennedy Airport for one hour. Uber was invited to join this protest but did not do so. Instead, the company posted a message on Twitter saying it had turned off its surge pricing feature, which is a function that increases the costs of a ride during times of high demand. Uber’s opportunistic behavior entailed that 200,000 to 500,000 people deleted the app, and the hashtag #DeleteUber was spread widely around social media as the heart of the protest against Uber. Later in 2017, however, Uber remained the most downloaded ride-sharing app, showing that many Americans were still happy to continue giving their money to the company, even if it profited from a racist banning and undermined a protest by the taxi drivers of New York City. |
| *Sources:*  <https://www.nytimes.com/2017/01/31/business/delete-uber.html>  <https://www.washingtonpost.com/news/dr-gridlock/wp/2017/01/29/uber-triggers-protest-for-not-supporting-taxi-strike-against-refugee-ban/> |

**Pretest of Study 3**

## Objective

One indicator that might be strongly related to consumers’ perceived egregiousness is the media coverage of the companies’ misbehavior. We expect that with increasing media coverage egregiousness increases (heat-up phase). We also expect that after media stop reporting, perceived egregiousness is no longer stimulated and declines, as no critical mass is reached in terms of consumers boycotting or media covering the topic. Consumers fall back on initial arguments against boycotting (e.g., brand image, subjective costs) and return to their original consumption patterns. We conducted this study to test whether negative media reports and consumer perception of an offending firm covary over time. The study traces the case of the e-tailer described in Study 3.

## Sample and Measures

Using Google News aided in finding 134 news articles related to the landmark TV documentary. Content analysis included articles published between February 13, 2013 (the publication date of the original article), and June 1, 2013, as a cutoff date. To track down as many articles as possible published on the subject, we used the company name as the main keyword. Furthermore, because the main issue of the documentary pertained to the working conditions at the firm deemed irresponsible, we additionally searched for two more keywords deemed relevant in combination with the company name, namely “working conditions” and “security firm.” This approach yielded a total of 520 news articles. Articles that did not directly relate to the topic—for example, dealing with new products or business reports of the company instead—were excluded, resulting in a salient set of 134 articles. Using frequency analysis to examine the valence of the articles (positive/neutral or negative), two coders analyzed the set of 134 news articles according to their tonality. Articles coded as having a positive tonality downplayed the severity of the events. Moreover, these articles emphasized that the situation applied only to a single location of the company and emphasized that working conditions were much better at other locations. In addition, positively toned articles included employee interviews to the point that the working conditions were not as deplorable as described. In contrast, articles coded as having a negative tonality echoed the negative content of the documentary or provided new information portraying the company in an unfavorable light. Across categories, the average intercoder reliability was sufficiently high (Krippendorff’s α = .63; Scott’s π = .63, Cohen’s κ = .63; percent agreement coefficient = .88; Lombard, Snyder‐Duch, and Bracken 2002).

## Results

Frequency analysis suggests that media coverage was intense during the first three weeks after the documentary was published, and peaked at the end of the second week. After the peak, the number of news articles on the event decreased continuously until it ceased completely after week 13. Regarding tonality, the majority of articles exhibited a negative tone during the first three weeks. Only a few articles had a positive tonality, including reports of employees enjoying favorable working conditions or news about positive company feedback (week 1: 5 positive articles vs. 32 negative articles; week 2: 9 positive vs. 35 negative; week 3: 4 positive vs. 12 negative). During the following three weeks, no positive articles were found, while the number of negative articles decreased (see Figure A1).

**Figure A1.** Dynamic Effects

**Notes.** Valence of news articles – positive valence: number of published articles with positive tonality (e.g., exaggerated severity of the reported situation); negative valence: number of published articles with negative tonality (e.g., more details about the companies’ conducted misbehavior); aggregated valence: difference between articles with positive valence and negative valence.
